# Implementation of Recycling Cigarette Butts in Lightweight Bricks and a Proposal for Ending the Littering of Cigarette Butts in Our Cities

**DOI:** 10.3390/ma13184023

**Published:** 2020-09-10

**Authors:** Abbas Mohajerani, Siu Qun Hui, Cary Shen, James Suntovski, Glen Rodwell, Halenur Kurmus, Marven Hana, Md Tareq Rahman

**Affiliations:** School of Engineering, RMIT University, Melbourne 3000, Australia; s3543776@student.rmit.edu.au (S.Q.H.); s3323107@student.rmit.edu.au (C.S.); s3546552@student.rmit.edu.au (J.S.); glenaidanrodwell@gmail.com (G.R.); s3432918@student.rmit.edu.au (H.K.); s3602822@student.rmit.edu.au (M.H.); s3631252@student.rmit.edu.au (M.T.R.)

**Keywords:** cigarette butts, recycling, fired clay bricks, bacteriological investigation, waste management, cigarette butt littering

## Abstract

Our cities, parks, beaches, and oceans have been contaminated for many years with millions of tonnes of unsightly and toxic cigarette butts (CBs). This study presents and discusses some of the results of an ongoing study on recycling in fired-clay bricks. Energy savings: the energy value of CBs with remnant tobacco was found to be 16.5 MJ/kg. If just 2.5% of all bricks produced annually worldwide included 1% CB content, all of the CBs currently produced could be recycled in bricks, and it is estimated that global firing energy consumption could be reduced by approximately 20 billion MJ (megajoules). This approximately equates to the power used by one million homes in Victoria, Australia, every year. Bacteriological study: CBs were investigated for the presence of ten common bacteria in two pilot studies. *Staphylococcus* spp. and *Pseudomonas aeruginosa* were detected in fresh used CB samples, and *Listeria* spp. were detected in old used CB samples. All of the CB samples except the dried sample had significant counts of *Bacillus* spp. Some species of the detected bacteria in this study are pathogenic. Further confirmation and comprehensive microbiological study are needed in this area. The contact of naphthalene balls with CBs had a significant disinfecting effect on *Bacillus* spp. The implementation procedure for recycling CBs in bricks, odour from Volatile Organic Compound (VOC) emissions in CBs, sterilization methods, CB collection systems, and safety instructions were investigated, and they are discussed. Proposal: when considering the combined risks from many highly toxic chemicals and possible pathogens in cigarette butts, it is proposed that littering of this waste anywhere in cities and the environment be strictly prohibited and that offenders be heavily fined.

## 1. Introduction

Cigarette butts (CBs) are the most common type of waste material discarded in the world. In 2016, an estimated 5.7 trillion cigarettes were consumed around the globe [1,2]. While, in Australia, 24 billion cigarettes were consumed, of which seven-billion were disposed of incorrectly [3]. That is, up to two-thirds of every smoked cigarette is littered to the environment [4]. Numerous countries have implemented strong tobacco control regulations, and a significant decline in cigarette consumption has been observed [5]. Despite this, it is estimated that the number of tobacco smokers is set to increase by seven- and 24-million in Nigeria and Indonesia, during the period 2015 and 2025 [1]. This is due to further growth in the market and population growth [6].

There are over 4000 chemicals present in a cigarette, seventy-two of which are known to be cancer-causing carcinogens [7,8,9]. The main toxic agents include carbon monoxide, argon, aromatic hydrocarbons, hydrogen cyanide, phenol, nitrogen oxides, formaldehyde, acetaldehyde, acetone, benzene, ammonia, and pyridines [10]. The filter of a cigarette is made of cellulose acetate fibers, whereby the filter modifies the particulate smoke components through particle retention [11]. However, cellulose acetate filters have poor biodegradability and they can take up to 10 years to decompose under normal environmental conditions [12]. Therefore, when CBs are freely dispersed in the environment, they pose a critical problem in terms of toxic waste for the urban and aquatic life [13,14,15,16]. Aquatic animals regularly consume CBs mistaking it for food and they have been found in the stomachs of fish, birds, sea turtles, and other creatures, leading to serious digestive issues [17].

As the world’s population increases, the number of CBs being littered is likely to rise drastically. In Australia, CBs were reported as the most common source of rubbish collected, representing 91.5% under the miscellaneous category [18]. As a result, it is becoming more imperative that an effective solution for this environmental problem be found and implemented.

Torkashvand and Farzadkia (2019) focused on developing control models for CB littering, while Marinello et al. (2019) analysed possible recycling techniques for CBs and evaluated the disadvantages and advantages of those methods studied [14,19]. In contrast, Kurmus and Mohajerani (2020) reviewed key research studies on the recycling of CBs and investigated their toxicological properties [20]. The paper explored the effectiveness, sustainability, and efficiency of those recycling methods. A major recycling method reviewed involved the development and the use of encapsulated CBs in asphalt concrete with acceptable physical and mechanical properties [21,22]. Furthermore, CBs have been used in several applications in small scales in order to produce items, such as pillows, plastic furniture, and shipping pallets. However, the credibility and sustainability of any method and product for recycling CBs must be investigated in terms of toxicity, leachability, and life cycle analysis.

Previous studies conducted by Mohajerani et al. (2016) tested the physical and mechanical properties, emissions, and energy savings of fired clay bricks incorporated with CB content, and presented promising results. The proposal was to incorporate 1% CB content by weight in 2.5% of the world brick production to solve a global waste catastrophe [23].

However, further testing and analysis are required for the brick manufacturing industry and government organizations to confirm that recycling CBs in fired clay bricks is a feasible and beneficial proposal. The objective of this study is to present and discuss some of the results of an ongoing research on recycling CBs in fired-clay bricks. It includes the calorific value of CBs and energy savings, laboratory study on the manufacturing and properties of bricks containing CBs, CB incorporation in the brick manufacturing process on an industrial scale, pilot bacteriological investigations of CBs, and possible CB odour reduction and sterilisation methods.

## 2. Calorific Value of Cigarette Butts and Energy Reductions

The incorporation of organic matter in clay mixtures assists in the firing process of clay bricks and, hence, the recycling of waste materials in fired clay bricks is an area of wide ranging research [24,25]. The calorific value of CBs must be considered to determine the energy saving benefits of the addition of CBs in bricks. The calorific value of a material determines the amount of heat (energy) that is produced by the complete combustion of that material. Materials with high calorific values (e.g., petrol) produce more energy when combusted than materials with low calorific values (e.g., wood). Consequently, utilising a high calorific value material as a partial replacement in a mixture with a low calorific value will reduce the overall required firing energy for bricks, as, when compared to a standard mixture, additional energy is produced by the materials’ constituents when combusted.

An investigation into the calorific value of cigarette butts was undertaken. Samples of used CBs with remnant tobacco and used CB filters were tested with the aim of determining the calorific value of each. The results showed that the average gross calorific value of CBs with remnant tobacco (representing the typical used CBs found discarded on the ground) was 16.53 MJ/kg. A higher value of 16.99 MJ/kg was recorded for the cellulose acetate filter in isolation. However, the lower value of 16.53 MJ/kg has been used in this study. The cellulose acetate content from CB filters reduces the required firing energy for clay bricks, due to it possessing a higher calorific value than clay [23]. The energy that was required for the successful firing of bricks was taken as 2 MJ/kg, as it lies within the range of values stated by Prasetsan (1995) and other investigators [26].

By utilising the tested CB calorific value and Equations (1)–(4), the estimated energy savings from the incorporation of 1% CB content into clay bricks is 9.3%.

Energy required to fire control brick (MJ),

(1) $$Q_{1}=q\times m_{1}$$

Energy required to fire CB brick (MJ),

(2) $$Q_{2}=q\times m_{2}-\mathrm{CV}\times m_{3}$$

Energy saved (MJ),

(3) $$Q_{1}-Q_{2}=q\times m_{2}-\left(q\times m_{2}-\mathrm{CV}\times m_{3}\right)$$

Energy Saved (%),

(4) $$\Delta E=Q_{1}-Q_{2}/Q_{1}\times 100\%$$

where

$m_{1}=$ mass of control brick (no CB content)
$m_{2}=$ mass of clay in brick containing CBs
$m_{3}=$ mass of CBs in brick
q= 2 MJ/kg energy required for firing clay
CV = Measured calorific value of used CBs, 16.53 MJ/kg.

It is estimated that, if 2.5% of all the bricks produced annually around the world include 1% CB content, the energy consumption of the process can be reduced by approximately 20 billion MJ (from the calorific value of 1% CBs with 9.3% energy saving) [23]. This approximately equates to the power used by one million homes every year in the State of Victoria, Australia. Therefore adding 1% CBs (about 20 kg/m^3^) to the brick clay results in significant energy savings, solves the CB pollution globally, and tangibly reduces the environmental impact of the brick manufacturing industry whilst providing financial savings to manufacturers through the reduction of energy for firing bricks.

## 3. Laboratory Study on the Manufacturing and Properties of Bricks Containing CBs

### 3.1. Manufacture of Samples

Cigarette butts (CBs) of varying sizes and brands were collected from dry bins and supplied by Butt-Out Australia Pty Ltd. for this study. PGH Bricks and Pavers (Victoria) provided the brick soil, which is a brown silty sandy clay. The geotechnical parameters of the soil were determined according to AS/NZS 1289.5.1.1:2003 and they are presented in Table 1 [27].

The manufacturing process of the bricks first involved drying the soil and CBs (if any) in an oven for 24 h at 105 °C. The soil was taken out of the oven and crushed into finer particles to pass through a sieve size of 2.38 mm. The soil and oven dried CBs were then placed inside a 20L capacity Hobart mechanical mixer and mixed for a total of 25 min. The calculated water content of 15.5% was gradually added during the mixing process. After the mixing was complete, the mixtures were sealed in airtight plastic bags and then left to settle for a period of 48 h to uniformly distribute moisture throughout the mix.

The next step was to compact the mixes while using a Servopac Gyratory compactor with a pressure of 240 kPa. A cylindrical mould was used during the compaction process. The moulds were 100 mm in diameter, in which a predetermined mass of soil was placed inside the mould in order to yield bricks with heights of approximately 50 mm.

After compaction, the samples were air dried for 24 h before being placed in an oven for 24 h at 105 °C. After being removed from the oven, the samples were fired in an electric furnace at 1020 °C for 3 h. The height, diameter, and weight of the bricks were measured while using a digital caliper and electronic scale at each stage of the manufacturing process to compute the diametric and height shrinkage of the bricks.

**Figure 1:**
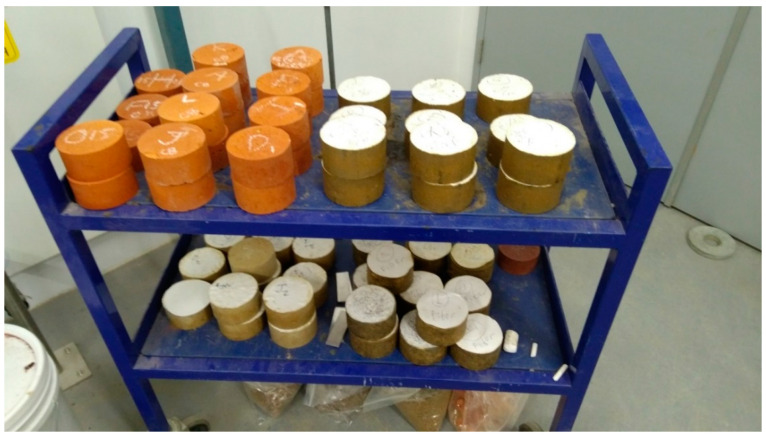
Bricks before and after firing.

The bricks were tested according to AS/NZS 4456.1:2008 after they were removed from the furnace (Figure 1) and allowed to cool to room temperature [28]. The compressive strength, initial rate of absorption (IRA), cold water absorption, shrinkage, and density were computed. Compressive strength tests were conducted on three samples from each mix using a Material Testing Machine (MTS), while the cold-water absorption and IRA testing were completed using a water bath.

### 3.2. Properties of the Bricks

Table 2 shows the average properties of the brick samples tested. The control samples containing no CBs and 15.0% moisture content were manufactured as a benchmark for further results. An additional 0.5% moisture content was added to the first batch of 1% CB bricks to account for the effect of the CBs, which could absorb moisture from the soil and, thereby, result in an undesirably dry mix. The optimum moisture content was used in order to achieve the maximum density during the manufacturing of the samples.

As expected, the addition of 1% CBs resulted in a reduction of the compressive strength to 27.49 MPa for the 15.5% moisture content sample and 25.77 MPa for the 17.5% moisture content sample. The common compressive strength for standard bricks is between 10 and 20 MPa. Varying moisture contents were used to investigate what effects this would have on the compressive strength and other properties of the bricks. Interestingly, shrinkage was reduced for the 15.5% moisture content samples, which could be attributed to the absorption of moisture by the CBs prior to firing. The whole CBs present in this sample may have also provided a reinforcing effect to resist shrinkage. The shrinkage of the 17.5% moisture content bricks was very similar to that of the control, thereby indicating that, for small samples, shrinkage is not greatly affected by the inclusion of 1% CB content. Eliche-Quesada et al. (2012) similarly reported a minor decrease in shrinkage with the addition of biodiesel production residues in fired clay bricks [29].

Cold water absorption increased with a decrease in density from 2134 kg m^−3^ to 1964 kg m^−3^ (8%). The initial rate of absorption was also higher for the CB bricks. Once again, this is expected, as a reduction in density relates to an increase in pore volume and, hence, a more absorptive material. Similar observations were reported in previous studies of bricks incorporating recycled paper processing residues [30]. The large initial rate of absorption for the 15.5% moisture content CB bricks can be explained by the voids that are left by the CBs after firing. Consequently, for both aesthetic and physical reasons, manufacturers should ensure that the CBs are completely disintegrated and mixed evenly before firing. It is important to note that the water absorption and initial rate of absorption values obtained to satisfy the relevant masonry standards for low-medium rise structural bricks. Finally, the thermal conductivity of the bricks decreases with the corresponding reduction in density, 1.107 W m^−1^ K^−1^ to 0.873 W m^−1^ K^−1^, a reduction of up to 21%. This is a fantastic result, as a lower thermal conductivity will reduce the amount of heat that the brick transfers. A house built with 1% CB bricks will use less energy for heating in winter and less energy for cooling in summer. This reduction in operational energy will result in huge global energy savings above what has been estimated purely from the reduction in embodied energy due to the calorific value of CBs that contribute to the firing process. The thermal conductivity was calculated while using Equation (5).

(5) $$TC=0.0558e^{0.0014D_{d}}$$

Where,

*TC* = thermal conductivity (W m^−1^ K^−1^)
*D_d_* = dry density of clay bricks (kg m^−3^).

These results confirm the findings in Mohajerani et al. (2016) [23,31,32]. Investigations into the leachates from manufactured CB bricks have already been conducted [23]. The Australian Bottle Leaching Procedure (ABLP) and the Toxicity Characteristics Leaching Procedure (TCLP) were both used on crushed bricks and solid bricks [23]. The testing was conducted on bricks containing 2.5, 5.0, 7.5, and 10.0% CB content. It was found that heavy metal concentrations were well below the levels stipulated by the USEPA and the Environmental Protection Agency of Victoria for concentrations of 10% and lower CB content [33,34]. Additionally, a comprehensive leachate study on leachate analysis of heavy metals in cigarette butts and bricks incorporated with cigarette butts has been carried out based on ABLP (2019) and USEP (2013) [21].

## 4. Brick Manufacturing Processes—Industrial Scale

### 4.1. Automatic Processes

The manufacturing process for clay bricks can be summarised by the following sequential processes: mining of raw materials, grinding, screening, blending (where desired mixes are formed by adding admixtures/other types of soil), forming, shaping, drying, firing, cooling, and packaging/shipment of the material [35].

#### 4.1.1. Stage 1. Winning

The raw materials that were used in the production of clay bricks include surface clays and shales, which are commonly acquired from open pit quarries [35]. Clay is the most commonly utilised material in the production of bricks. Approximately 96% of all bricks manufactured in the United Kingdom are produced with clay [36]. Typically, clay will be extracted in bulk quarries on a few occasions per year and stockpiled (bulk winning), which allows extraction to occur during good weather [37]. Quarries are sometimes located on-site alongside manufacturing facilities, whilst other facilities obtain raw materials via road or rail transportation [35]. Raw materials for brick making are stockpiled on-site, usually in the open, where rainwater washes out some salts that could cause efflorescence. These stockpiles may contain a sufficient volume of raw material to satisfy up to a year of brick production [36].

#### 4.1.2. Stage 2. Primary Crushing

Raw materials are then fed into a primary crusher for initial size reduction, typically by a truck or front-end loader [35].

#### 4.1.3. Stage 3. Blending

The minerals within the clay are responsible for the bricks’ final properties, such as colour [36]. Consequently, different clays and admixtures are combined at this early stage to produce a mix design that will achieve the desired properties of the final product [37]. Throughout this stage, water is added consistently, where the water, brick clay soil, and any additional additives are mixed together. The blending process also reduces the size of the materials within the mixture.

#### 4.1.4. Stage 4. Grinding

The material, now initially reduced in size, is typically fed into a secondary crushing mechanism. Manufacturing plants typically utilise a pan mill for secondary crushing, which can be used with both dry and moist clay mixtures [38]. The plant that is used for grinding is generally heavy, creates a lot of vibration, and, unlike blending, the mixture at this stage is extremely difficult to assess. The brick soil mix at this stage is very fine [37].

#### 4.1.5. Stage 5. Screening

From here, the material is screened to ensure that the correct size reduction has been achieved. Oversized clay particles are returned to the secondary crushing system [38]. The material is then conveyed either to storage areas (such as stockpiles or silos) or directly into a mill room for forming.

#### 4.1.6. Stage 6. Forming and Shaping

Extrusion, also known as the stiff-mud method, involves producing a mixture with the desired plasticity by adding 10–15% water content. Once the desired water content (WC) has been reached, and the mixture is homogenous, it is passed through a vacuum chamber to de-air the mixture. De-airing removes pore air from the mixture, increases the plasticity, and contributes to the final product strength. Finally, the clay is extruded to produce a ‘column’ of clay, which is then coated if required and cut into bricks by a set of automatic cutting blades. Around 90% of all bricks produced in the United States of America are produced via extrusion [39].

Moulded.

The moulded brick process, otherwise known as the soft-mud process, is suitable for clay with water contents too high to be used in extrusion. Instead, a mixture with approximately 20–30% water content is formed. This mixture is placed and compacted in brick moulds to prevent the mix from sticking to sand or water. This method can be utilised by machine or hand [39].

Dry-Press.

This process is used for very low-plasticity clays. A mixture with up to 10% water content is created, which is then compressed by air or hydraulic rams into steel moulds [39].

#### 4.1.7. Stage 7. Drying

Once the bricks have been formed, they are typically stacked onto kiln cars and sent into a holding area where they are later loaded into a dryer. Most dryers utilise waste heat from the cooling zone of a kiln, and they are typically heated to 204 °C; however, some manufacturing plants may choose to use fuels, like gas, to heat up their driers [35]. It should be noted that, in some instances, air drying of bricks is utilised; however, this method is avoided where possible due to weather impacts, increased duration of production, and uneven drying. Two of the most common driers used are tunnel dryers, in which a kiln car will slowly move through a heated tunnel, and chamber dryers, where bricks are loaded onto pallets inside a large chamber [38].

#### 4.1.8. Stage 8. Firing

The bricks are then fired in kilns. There are two main types of kiln—intermittent kilns and continuous kilns. Intermittent kilns, such as clamp and down-draught, must be allowed to cool before bricks can be unloaded and reloaded. They are typically only utilised where special colour effects are required [36]. Continuous kilns are used in large scale production and they are capable of firing bricks at a steady rate due to the continuous loading, firing, and unloading process [37]. The most popular type of continuous kiln is a tunnel kiln, where bricks are loaded into kiln cars and pass through various carefully monitored temperature zones inside a tunnel. The advantage of this method is that the process is continuous. These tunnel kilns can range from 104 to 152 metres in length and they can reach a maximum temperature of approximately 1090 °C in the firing zone.

#### 4.1.9. Stage 9. Unloading, Packaging and Shipping

After successful firing, bricks enter a cooling zone (if continuous kiln), where they can cool to near ambient temperatures. They are then removed from the kiln, stored, packaged, and shipped [35].

### 4.2. Manual Processes

Clay bricks have been utilised throughout history by many civilisations. Bricks were used in the construction of the city of Babylon about 6000 years ago [36]. Although the industrial revolution introduced machine processes into brick manufacturing, in some places around the world, bricks are still made by hand [36].

In some nations, such as India, brick manufacturing is predominately a manual process, where traditional processes of making bricks have changed little over time [40]. The typical process can be described, as follows:

#### 4.2.1. Stage 1. Winning

Clay is mined (either by hand or mechanical means) and stored in piles in the open. Rainwater is said to make the clay soft and removes unwanted chemical oxides from the clay.

#### 4.2.2. Stage 2. Mixing

When ready, water is added and mixed with the clay until a mixture with enough plasticity for moulding is created. The mixing process is conducted with hands and feet.

#### 4.2.3. Stage 3. Moulding

Once the desired consistency has been achieved, the workers roll a large sample of the mixture in sand, before being pushed into steel brick moulds. The sand acts as a lubricant that stops the mixture sticking to the mould.

#### 4.2.4. Stage 4. Drying

These moulds are placed into a drying area where sun has access to the moulds. The moulds are occasionally turned over to prevent non-uniform drying and consequential warping of the bricks.

#### 4.2.5. Stage 5. Firing

After a sufficient drying process (typically up to two weeks), the bricks are arranged in a kiln and fired. Once firing is completed (decided at the discretion of the brick maker), bricks are sorted according to colour [40].

#### 4.2.6. Stage 6. Cooling

Bricks are unloaded from the continuous kilns, where they can cool to ambient temperatures. In the case of intermittent kilns, the entire kiln and all of the bricks it contains can cool in between firing cycles. Bricks are unloaded once the entire system has cooled.

#### 4.2.7. Stage 7. Packaging and Transportation

The bricks are sorted, depending upon the qualities, such as colour, and then packaged. These bricks may then be transported off-site, utilised in the construction of new kilns or re-fired (if sufficient firing has not occurred) at the discretion of the plant manager.

It is important to note that, whilst the general method of manual brick manufacture remains the same, regional variations are common, which accommodate factors, such as local geography, climate, etc.

In Africa and South America, large fires fuelled by wood are used to heat kilns, which are then insulated with grass or mud. In nations. such as India and Mexico, large volumes of bricks are fired at once, and the thermal mass is used for insulation. Fuel for kilns can be wood or coal, but, in some cases, where these are not accessible, garbage and biomass can also be used [40].

## 5. CB Incorporation into the Automatic and Manual Process

### 5.1. Incorporation into Automatic Processes

Three methods are suggested to incorporate the CBs into the automatic manufacturing process: Method 1—Addition of whole CBs with no modification, Method 2—Addition of pre-shredded CBs, and Method 3—Addition of a concentrated CB pre-mixed brick clay (with a high % of CB content).

Although three methods are suggested, for the most part, the methods share a common sequence. Once CBs have been delivered on-site, they will be stored, cleaned, and handled strictly according to the Occupational Health and Safety (OH&S) requirements. 

#### 5.1.1. Method 1—Addition of Whole CBs with No Modification

**Figure 2:**
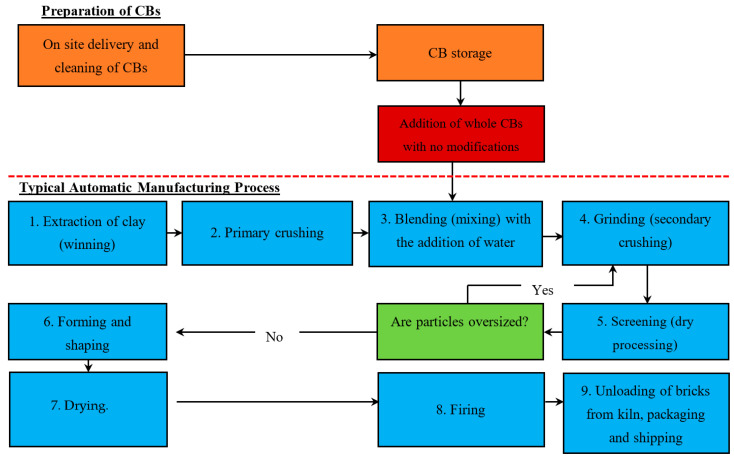
Method 1—The addition of whole cigarette butts (CBs) into the Automatic Manufacturing Process.

Figure 2 demonstrates the stages of the Automatic Manufacturing Process and the proposed stage to add the whole CBs with no modification. This paper suggests that whole CBs should be added during stage 3, the blending stage, with the addition of water. When CBs are introduced during this stage, the cohesive characteristics of the brick clay mixture and the addition of water reduce or even negate any potential airborne particles that may derive from the CBs. Adding CBs during stage 3 also reduces the initial size (disintegration) and allows for CBs to be better distributed within the mix. Improvements in quality can be achieved with longer mixing times [23].

#### 5.1.2. Method 2—Addition of Pre-Shredded CBs

**Figure 3:**
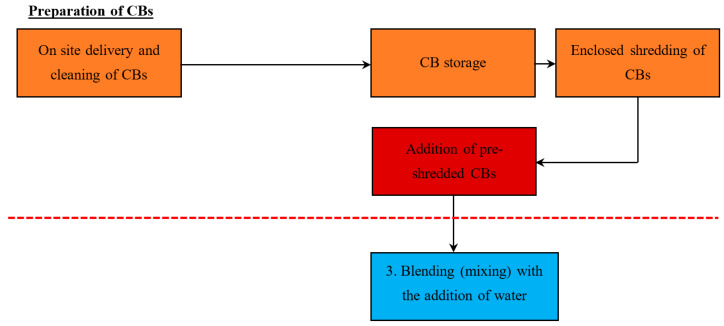
Method 2—Addition of pre-shredded CBs into the Automatic Manufacturing Process.

Figure 3 demonstrates the proposed stage to add the pre-shredded CBs. As the preparation process is different, refer to Figure 2 for the common stages. This paper suggests that pre-shredded CBs should be added during stage 3, the blending stage, with the addition of water. When introducing the CBs to stage 3, it is important to minimise the exposure of the CBs to open air environments, as chemicals within the CBs may diffuse into the air with other dust particles. Exposure to this air could be a safety risk for nearby personnel. A pipeline pathway between the enclosed shredding system and blender is recommended in order to minimise environmental pollution, as the shredded CBs are directly incorporated in stage 3. Water is added throughout the blending stage, as moisture within the mixture entraps chemicals and mitigates potential airborne particles.

It is important to understand that the actual shredding of the CBs must be performed in a fully enclosed environment, such as an enclosed industrial shredder. This is necessary, as it keeps the dust and chemicals from spilling out into the environment. Enclosed shredding minimises environmental pollution and the exposure of personnel that could occur when compared to shredding in an open air environment. When compared to method 1, as the CBs are already pre-shredded, it is expected that there will be improved uniformity in particle size and distribution within the mix, leading to clay bricks with more uniform properties.

#### 5.1.3. Method 3—Addition of a Concentrated CB Pre-Mix (with a High % Content of CB)

**Figure 4:**
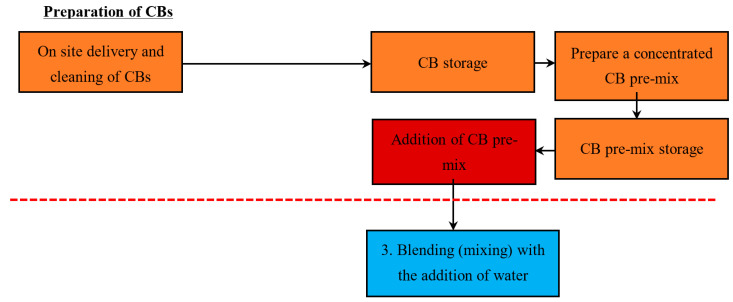
Method 3—Addition of a concentrated CB pre-mix into the Automatic Manufacturing Process.

Figure 4 demonstrates the proposed stage to make and add the concentrated CB pre-mix. As the preparation process is different, refer to Figure 2 for the standard stages. This paper suggests that the concentrated CB pre-mix should be added during stage 3, the blending stage, with the addition of water. The concept of this method allows for clay brick manufacturers to make a CB pre-mix and store it until needed for specialised brick orders. This means that manufactures can create different types of brick products that contain varying CB content, which can be made to order by the client. This pre-mix consists of a high content of CBs, water, and soil. The premix is then incorporated during the blending stage into the main line of bricks being produced. Manufacturers should take care when adding the premix into blending in order to ensure that the high concentration CB content mix is diluted to the desired CB content outcome.

An issue with this method is that it may require another conveyor/plant to transport the pre-mix into stage 3. This suggestion requires a capital investment, which should be considered before implementing it into the production line.

#### 5.1.4. Appropriate Personal Protective Equipment for the Automatic Manufacturing Process

There is a possibility when dealing with CBs that the chemicals that are present in CBs may be distributed into the air with other dust particles. Therefore, it is essential that all workers exposed to excessive dust particles be equipped with dust masks. If moisture is added to the mixture at the same time as the introduction of CBs, then the risk of inhalation of airborne particles is drastically reduced, or possibly mitigated, as mentioned in the above methods. However, there is the potential for leachates contaminating surfaces, such as conveyor belts, and mills, etc., due to the potential for the moisture to introduce metals to the environment [41]. As a result, workers encountering this mixture or associated surfaces should also wear protective gloves and long-sleeve clothing.

#### 5.1.5. Odour Issues and Reduction Methods for the Automatic Manufacturing Process

Odour can be a significant issue that is introduced through the incorporation of CBs into the automatic brick making process. Odour is caused by the diffusion of chemicals that are entrapped within CBs being released, such as volatile organic compounds (VOCs) [42,43,44]. Odour can be displaced by air current or diffusion, both of which rely upon the environmental conditions and time. The smell is a biochemical reaction, where VOCs attach themselves to the olfactory receptors that are located in the nose. These receptors then send an electrical signal to the brain that is perceived as odour [45]. Not all VOCs have an odour associated with it, for example, methane, which is an odourless chemical [46]. Due to smell being the human perception of the physical world, it is hard to physically or chemically measure and quantify smell, as even the smallest amount of VOC can produce an odour. This is a problem, because CBs are introduced in the blending stage, but the odour can persist until firing of the CB bricks occurs (stages 3–7).

Odours themselves contain four attributes that humans can detect and describe. These attributes include the detection threshold, intensity, character, and hedonic tone. The detection threshold is the minimum amount of chemicals needed to detect odour; intensity is how strong the odour is upon and after detection; character is defined as what the odour smells like; and, hedonic tone is how pleasant the odour is to smell [47]. Out of the four attributes, only two of them are quantifiable—detection threshold and intensity. The detection threshold is defined as the point at which 50% of the population (normally a panel of six people) can detect an odour and is recorded numerically as one odour unit per metre cube $\left(1\;o.u./m^{3}\right)$ [48]. The intensity of an odour can be quantified using the Weber–Fechner law, in which an increase in concentration is perceived as a large increase in intensity when the concentration value is small. The equation intensity versus concentration is a logarithmic relation (Equation (6)), which means that, for CBs in the brick manufacturing process, a small increase in CB content can lead to a large increase in the intensity of the CB odour [48].

Intensity versus CB% curve,

(6) $$I=K_{w}\ln\left(\frac{CB}{CB_{0}}\right)$$

where;

I= Intensity of CB Odour
$K_{w}=$ Constant based on how sensitive the human nose is at detecting odour from CB
$CB_{0}=$ Cigarette butt content (%) to which 50% of the population can detect an odour (AMOL)
CB= Cigarette butt content.

#### 5.1.6. Controlling Odour Level

The potency of the odour is dependent on the volume of chemicals present in the brick factory. This would be the rate of diffusion of the volume of CBs present, which will vary according to the ventilation rate [43]. Every brick factory has its own unique ventilation rate, which is based on the environmental conditions and design of the factory [25]. The ventilation rate will fluctuate a little, but will remain as a constant and, therefore, it is recommended to change the CB content, as it is an independent variable subject to change by the manufacture. The process of changing the CB content within the mix should be an incremental one, where manufactures start with a small number of CBs then assess the outcome. From there, if it is found that the Acceptable Max Odour Limit (AMOL) has not been reached, then the CB content should be increased by a small amount. The process should then be repeated until AMOL is reached. The Acceptable Max Odour Limit (AMOL) is defined as the cigarette butt content, at which 50% of the workers surveyed can detect an odour. Once AMOL is reached, it is recommended to use a previous CB increment. This solution can also be implemented in conjunction with the other solutions that are suggested below.

Increasing the ventilation rate by installing exhaust fans or fume extractors at specific points along the production chain is a viable solution. The odour can either be vented or captured using filters (such as activated carbon). Unlike controlling the CB content in the mix, increases in ventilation require some capital to be invested. The benefit of this solution is that it provides a direct and permanent increase in odour reduction within the factory.

#### 5.1.7. Odour Elimination Using UV Light

A possible elimination method for cigarette odour is to treat the odour with UV light, which could oxidise the odours that are emitted from the cigarette butts. It does this by shattering the molecular bonds through the impact energy and then allowing the elements to oxidise to form new molecules that are odourless or have less odour [49]. UVC light has the capacity to break down carbon hydrogen bonds, which have a bonding strength of 413 kj/mol. UVC with a wavelength of 254nm can be used to break down the bond due to the photon containing 470 Kj/mol of energy [49]. Oxidation can then be achieved by breaking down the dioxide molecules that are found within the air. UVV light is required in order to break down the oxygen bond, which has a bonding strength of 498 Kj/mol. UVV with a wavelength of 185 nm can be used to break down the bond due to the photon containing 645 Kj/mol, which is enough to shatter the bond [49].

### 5.2. Incorporation into Manual Processes

CBs in the manual brickmaking process should only be incorporated if handled with care, and strict OH&S procedures are followed, where workers always utilise the required PPE. The problem of odour should also be addressed, as discussed above.

The addition of CBs to manual manufacturing processes is quite simple, as the mixture is easily accessible and it is available at all stages leading up to the moulding and firing of the bricks. Two methods are suggested to incorporate the CBs into the manual clay brick making process: Method 1—Addition of whole CBs with no modification and Method 2—Addition of a concentrated CB pre-mix (with a high % content of CB).

Like the automatic manufacturing process, once CBs have been delivered on-site, they will be stored, cleaned, and handled strictly according to OH&S requirements.

#### 5.2.1. Method 1—Addition of Whole CBs with No Modification

**Figure 5:**
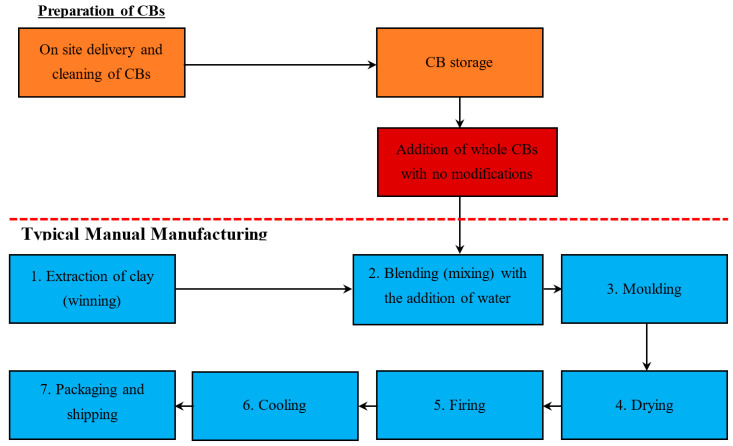
Method 1—Addition of whole CBs into the Manual Process.

Figure 5 demonstrates the stages of the Manual Process and the proposed stage to add the CBs. This paper suggests that whole CBs can be incorporated into the mixture between stages 2 and 3, after sufficient water has been added. This will reduce the risk of inhalation of dust particles containing any toxic chemicals from the CBs. Jrup (2003) states that environmental contamination by heavy metals predominately occurs via pathways leading to the air (such as processing of materials, combustion), soil (leaching into groundwater), and water (via surface runoff) [41]. As a result, it is expected that there will be no exposure to airborne contamination if the CBs are added when the mixture is wet.

#### 5.2.2. Method 2—Addition of a Concentrated CB Pre-Mix (with a High % Content of CB)

**Figure 6:**
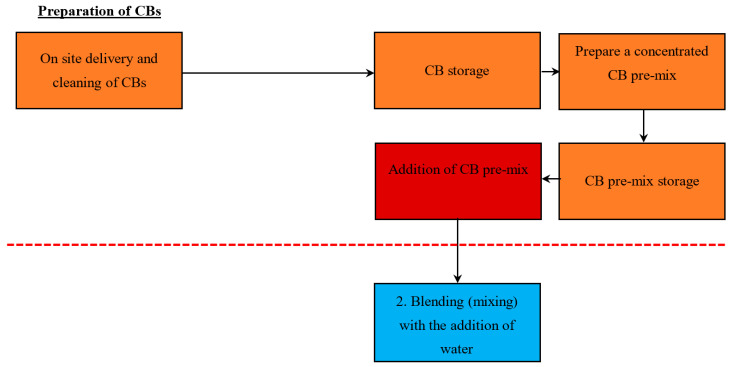
Method 2—Addition of a concentrated CB pre-mix into the Manual Process.

Figure 6 demonstrates the proposed stage to make and add the concentrated CB pre-mix. Refer to Figure 4 for the shared common stages, as only the preparation stage is different. This paper suggests that the concentrated CB pre-mix should be added between stages 2 and 3. This method may be more appropriate for the manual process, where the pre-mix can be stored in appropriate containers on-site and ready for use.

#### 5.2.3. Appropriate PPE for the Manual Process

The negative effects on human health from heavy metals are linked to lead, cadmium, mercury, and arsenic [41]. Studies have shown that three out of the four heavy metals stated above are present in CB smoke [9].

Therefore, it is essential that any worker who comes into direct contact with this moist mixture wears heavy-duty personal protective equipment (PPE). Examples of PPE include heavy-duty gloves and long-sleeve clothing for those mixing via hand and gumboots for those utilising their feet.

#### 5.2.4. Odour Issues and Reduction Methods for the Manual Process

As stated before, odour can be an issue in the production and use of CB bricks. In the manual process, odour is a problem in the moulding and drying stages (stages 3–4) [40]. The drying stage, which could last for up to two weeks, takes place in an open environment where the sun has access to the moulds. Gradually introducing CBs in the brick making process and assessing for AMOL would be an ideal low-cost solution. This is considering that the bricks would be placed in an open air environment, where the odour can be carried away by the air currents. However, if the environment is densely populated, the CBs should be treated using effective UV light or other methods before manufacturing. If UV treatment cannot be used, it is not recommended that CBs be incorporated into the manual brick making process.

## 6. Recycling CBs on an Industrial Scale: Collection and Processing

### 6.1. CB Collection Systems

**Figure 7:**
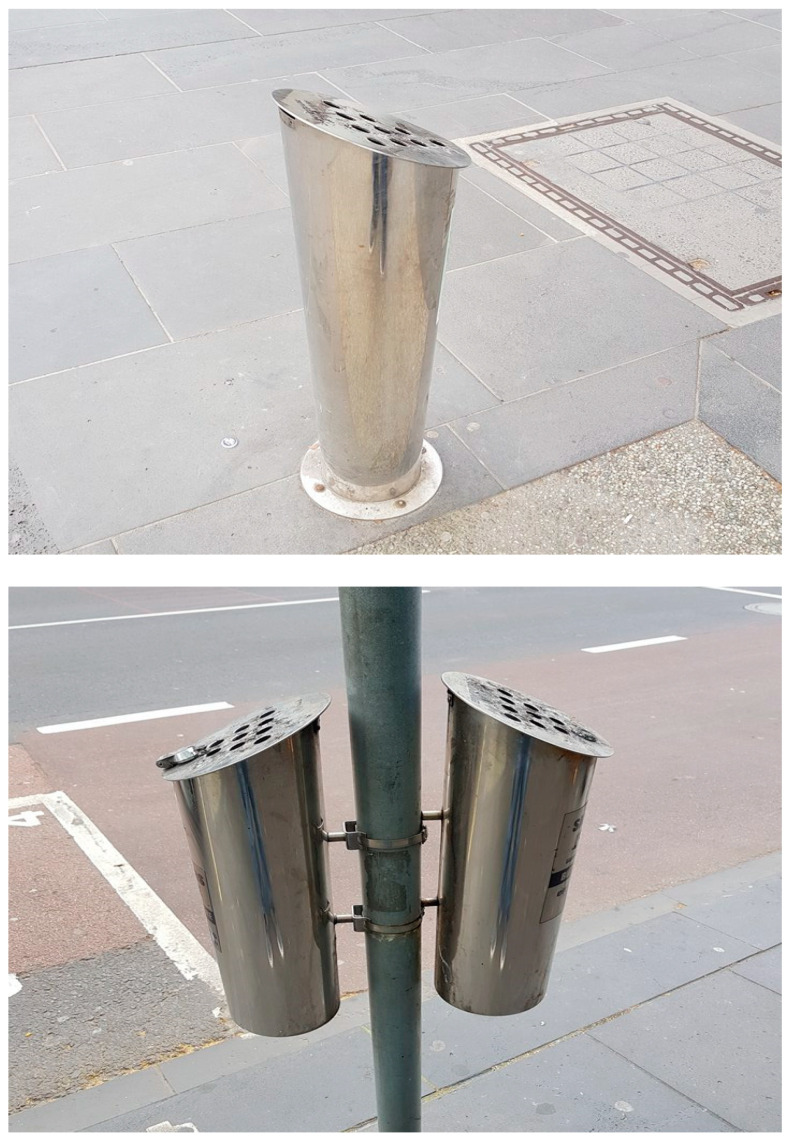
Typical CB Receptacles: free standing (**top**) and post mounted (**bottom**).

The huge environmental problem that is presented by CB litter has steadily gained focus across the world. Several companies in Australia have developed CB disposal systems in order to reduce the number of CBs being incorrectly disposed of. These companies provide receptacles (Figure 7) for the disposal of CBs, in addition to servicing regimes and the removal and final disposal of CB waste. Currently, much of this waste is placed in landfills or incinerated at the expense of the collection company. In all cases, clients (local governments and other organisations) can pay a periodic rental fee per receptacle, which includes the cost of collection, servicing, and final disposal. Receptacles take many forms, including wall-mounted, post-mounted, and free standing [50].

It is suggested that these companies deliver CBs (after appropriate preparations) directly to the brick manufacturing facilities, instead of landfill or incineration sites. The advantages of delivering CBs directly to brick manufactures are as follows: firstly, collection companies can save large amounts of money by not having to pay for incineration or landfill of CB waste. Secondly, the negative environmental effects of incineration and landfill can be avoided. Finally, brick manufacturers can gain access to existing collection systems without having to pay for services or having to implement their own collection schemes. However, it should be noted that, upon wide-scale introduction into bricks, CBs may be considered to be a valuable source of energy, and, when demand is sufficient, collection companies may decide to charge brick manufacturers for delivery. The safe storage and handling of used CBs on-site are discussed in the following section of this paper.

### 6.2. Preliminary Bacteriological Investigations

A serious concern in the recycling of used CBs is that they may be contaminated with bacteria or viruses from contact with people or the environment. Consequently, this study undertook two pilot investigations to determine whether the tested species of bacteria were present and if these bacteria posed a health risk to the waste collection and processing personnel.

The first pilot investigation focused on determining whether any of several common bacteria were present on either the used or unused cigarette filters. The purpose of this investigation was to determine if it was plausible that bacteria could be present and survive on discarded CBs. The second pilot investigation provided further results where additional species of bacteria were tested. The second test was conducted on used, unused (control), and dried CBs, as well as CBs that were in contact with two naphthalene balls (mothballs).

#### 6.2.1. Pilot Investigation 1

This investigation consisted of two samples and one control group: Control group (unused CBs); Sample 1—Used CBs, seven-day old sample; and, Sample 2—Used CBs, collected on the day sample.

**Figure 8:**
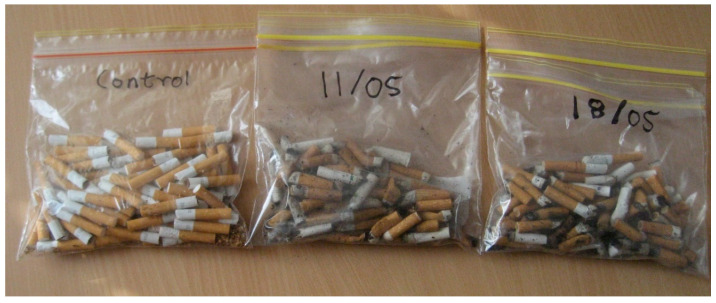
CB samples utilised for preliminary bacteriological study 1. The dates indicate when the used samples were collected in 2017.

The control group for the study consisted of generic cigarettes, which were bought and cut down to a length consisting of the filter plus 1.5 cm (for a total length of 3.5 cm). This was to ensure that the tobacco content of the control group closely resembled that of a smoked cigarette. A previous study has shown that some *Pseudomonas* spp. can thrive on the nicotine in solid tobacco waste (Wang et al. 2004). As a result of this finding, it was essential to ensure that similar volumes of tobacco were present in all of the samples (Figure 8).

The CBs used for this investigation were collected from the tops of bins around Victoria’s Melbourne Central Station. Collecting CBs from the bins increased the chances that the samples were fresh. Melbourne Central Station is a central hub for travel in Melbourne’s CBD and, therefore, it provides reasonably representative samples (i.e., people from many areas and socio-economic backgrounds travel through this area).

In this preliminary bacteriological investigation, the presence of *Salmonella* spp., *Escherichia coli*, *Pseudomonas aeruginosa*, *Enterococcus faecalis*, Coagulase + ve *Staphylococcus* spp., and *Streptococcus* spp. were investigated.

*Escherichia coli* live naturally in the intestinal tract of humans and animals and, in most cases, is harmless [51]. However, some *E. coli* are pathogenic and they can cause diarrhoea or other illnesses [51]. Although the most vulnerable include the elderly and young children, anyone can be infected. The bacteria can be spread through contact with unclean surfaces that are contaminated with faecal matter, such as human hands or door handles [51], and infections start when this bacteria is consumed. As a result, it is possible that *E. coli* can be spread to CBs from unwashed hands that have encountered contaminated surfaces, or they may even be present within the mouth when contaminated food is consumed. Thus, the presence of *E. coli* was investigated.

*Salmonella* spp. commonly causes gastro, and it is spread through the ingestion of improperly cooked food, water, or through hands that have encountered animal faeces [52]. As a result, it is plausible that *Salmonella* spp. could be present on used CBs if smokers have not washed their hands after coming into contact with animals, another infected person, or a contaminated material, such as improperly cooked food. As this bacterium enters the body through the consumption of contaminated food, it is also possible that it is present within the mouth.

*Pseudomonas aeruginosa* is primarily responsible for the infection of humans by *Pseudomonas* strains of bacteria. Healthy people who are infected generally only present mild symptoms, but those with a weak immune system (such as those who are already sick) can suffer severe infections that can be fatal. *Pseudomonas aeruginosa* can cause ear infections, eye infections, and skin rashes in healthy people, whilst people with a weak immune system may contract blood infections and pneumonia [53]. It is primarily spread by hand or from contact with contaminated equipment. *Pseudomonas aeruginosa* thrives in moist environments.

Most *Enterococcus* species are not pathogenic and they are naturally found in the intestinal tract of humans and animals. However, some strains, including *Enterococcus faecalis*, are known pathogens that can cause several illnesses, including urinary tract infections and infections of open wounds. *Enterococcus* species are difficult to kill, because they have developed a resistance to antibiotics, including penicillin [54]. From the intestinal tract, *Enterococcus* species could spread to surfaces, and hands, resulting in contamination, where it can be distributed through direct contact (WA n.d.). It is entirely possible that these bacteria could be spread to the surface of CBs through contaminated hands, hence it was investigated.

*Staphylococcus* bacteria are found naturally on the skin and in/around the nose (Healthdirect 2018). It is estimated that around one-third of humans carry *Staphylococcus* [55]. In most cases these bacteria cause no harm, but can become pathogenic if they enter the body. Its population growth can result in an infection. Serious issues include skin impetigo, wound infection, cellulitis, pneumonia, septic arthritis, sepsis, and endocarditis [56]. Some strains are particularly dangerous, as they are resistant to antibiotics. It is likely that contact with the skin around the mouth or the hands of people that have touched their nose could result in the contamination of CBs by *Staphylococcus* bacteria. As contaminated food enters the body, *Staphylococcus* bacteria may also be present in the mouth, which may be transferred to CBs.

*Streptococcus* bacteria are found in the upper respiratory tract and skin of humans. There are two types, Group A and Group B. Group A can cause the common ‘strep throat’, in addition to other illnesses, such as scarlet fever, impetigo, and cellulitis. Group B can cause blood infections, pneumonia, and meningitis [57]. The bacteria can be spread from person-to-person through sneezing and contact with contaminated surfaces. As a result, it was determined that these bacteria could be present on used CBs.

**Figure 9:**
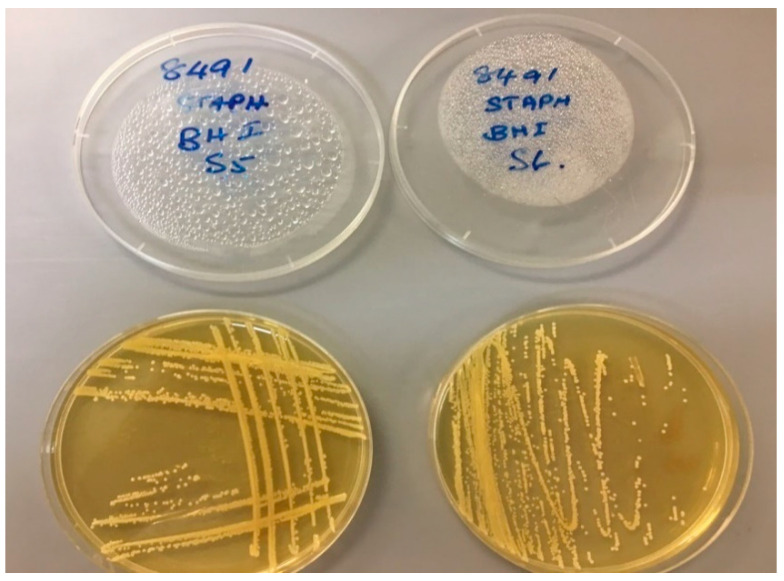
Confirmation of presence of *Staphylococcus* spp. in sample 2.

The results presented in Table 3 show that Coagulase + ve *Staphylococcus* spp. were sufficient in number in sample 2, whilst there were insufficient numbers to be detected in sample 1. The presence of Coagulase + ve *Staphylococcus* is shown in Figure 9.

This bacterium was not detected in sample 1, which could be an indication that *Staphylococcus* bacteria struggle to survive on CBs for an extended period. Alternatively, it could indicate that *Staphylococcus* was only present on the CBs of sample 2. Despite the confirmation of the presence of these bacteria, it has been reported that *Staphylococcus* requires a colony count exceeding 100,000 to infect humans [58]. However, it is possible that these bacteria could enter via the mouth or through cuts in the hands of workers, where the colony could flourish to an infectious concentration. Consequently, it is recommended that gloves should be used when handling CBs.

A very small concentration of *Enterococcus faecalis* was also detected in sample 1, which reinforces the prediction that unclean hands can transfer intestinal bacteria to CBs. It is possible that the original concentration was higher, and the bacteria had slowly died over time. It is also plausible that the original concentration was just as low and the bacteria can effectively survive on the surface of the CBs for a few hours. In either case, the concentration is a negligible one, but it shows that the bacteria are capable of surviving on the surface of used CBs for an unknown period.

#### 6.2.2. Pilot Investigation 2

**Figure 10:**
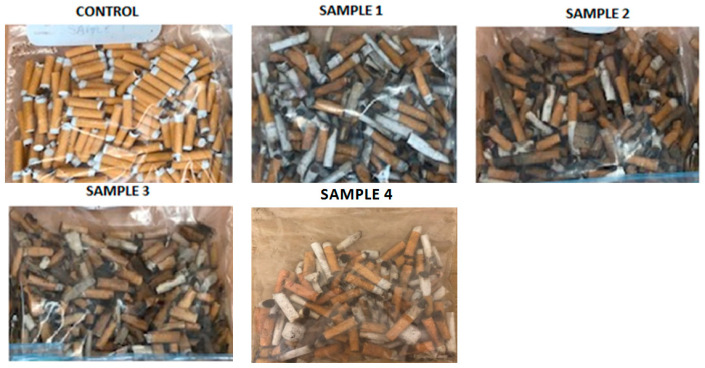
CB samples utilised for preliminary bacteriological study 2.

This investigation consisted of one control group and four additional samples (Figure 10): Control group (unused CBs); Sample 1—Used CBs, fresh CBs collected the day before the test; Sample 2—Used CBs, older samples; Sample 3—Used CBs, older samples that were dried at 105 degrees for 6 h; and, Sample 4—Used CBs, fresh CBs in contact with mothballs for 24 h.

Similar to the pilot investigation 1, the control group for pilot investigation 2 was prepared while using the same method and preparations. The sample size was approximately double when compared to investigation 1, as more tests were required to investigate the larger variety of bacteria. The control group was prepared a day before the test.

For sample 1, fresh CBs for this investigation were collected from the tops of bins and their tilt trays as well as ashtrays from restaurants/cafes around Melbourne’s CBD. Fresh CBs were collected in this manner in order to reduce the chance of the butts being very old and the chance that the butts encountered the ground. The samples were collected on the day before the testing. For sample 2, old CBs that were a few months old were collected from RMIT University storage. Sample 3 comprised old CBs that were a few months old and collected from RMIT storage; however, these CBs were placed in an oven at 105 degrees for 6 h. Sample 4 were fresh CBs that were collected in the same manner as sample 1, but had been treated with mothballs. The treatment consisted of placing two naphthalene (mothballs) balls inside a bag full of CBs and leaving for 24 h before removing them from the bag for testing. Samples 3 and 4 were two of the tested treatment methods for reducing the bacterium counts.

In this preliminary bacteriological investigation, the previous six bacteria were investigated: *Salmonella* spp., *Escherichia coli*, *Pseudomonas aeruginosa*, *Enterococcus faecalis*, Coagulase + ve *Staphylococcus* spp., and *Streptococcus* spp. Furthermore, an additional four types of bacteria were investigated: *Listeria* spp., *Legionella* spp., *Bacillus* spp., and *Clostridia* spp.

*Listeria* spp. are present in a wide variety of environments, including the soil, in animals, and in areas with water [59]. Most of the species of this bacteria group are not pathogens; however, there is one pathogen within this species that is harmful to humans, the ‘*Listeria monocytogenes*’ [59]. This pathogen is harmful, as it can cause listeriosis. Although most healthy people who are infected generally only present mild symptoms, it can prove fatal to people with a weak immune system, the elderly, and can cause pregnant women to undergo a miscarriage [59]. *L. monocytogenes* can exist and grow in food. Through consumption, *L. monocytogenes* may exist within the mouth, where it is possible that it may spread to CBs. Therefore, the presence of this species has been investigated.

*Legionella* spp. are naturally present in water sources of the environment. This pathogen is harmful when it grows within artificial water systems, such as pipes, which are common within households [60]. *Legionella* spp. can cause two main illnesses—pneumonia and Pontiac fever [60]. Most healthy people who encounter *Legionella* spp. are not affected. Those vulnerable include the elderly, smokers, and people with a weak immune system. *Legionella* spp. can enter the body via respiration and aspiration. It is possible that *Legionella* spp. may be present in the evaporated water that people can breathe if a colony is large enough and is thriving within an artificial water system [60]. Though less frequent, people may also get sick when drinking water contaminated with *Legionella* spp., as it could accidentally enter the lung [60]. It is possible that this pathogen is spread to CBs when exposed to a water environment and, thus, the presence of this species has been investigated.

*Bacillus* spp. are generally present in soil environments [61]. They are most commonly found in raw vegetables, water, organic matter, dust, and some flora species; however, there have been scenarios where *Bacillus* spp. was found in other types of food groups, such as meats and rice, and in hospital environments [61]. Most of these species are non-pathogenic. When *Bacillus* spp. contaminates food, it typically survives the cooking procedure and, as a result, food poisoning is likely to occur upon consumption [62]. This can be prevented through proper food handling. Gastrointestinal illness can develop if a high quantity of *Bacillus* spp. is consumed [62]. It is possible that *Bacillus* spp. could spread to CBs when exposed to soil, contaminated hands, contaminated surfaces, or the mouth, hence the presence of this species was investigated.

*Clostridia* spp. are mainly present in soil environments where organic matter exists [63]. Other locations include the sediments of aquatic environments, and the intestinal tract and normal microbial flora of animals and humans [64]. Pathogenic species that are harmful to humans include, but are not limited to, *C. botulinum*—which can cause food-borne botulism, *C. tetani*—which can cause tetanus, *C. perfringens*—which can cause wounds, surgical infections, gas gangrene, and food poisoning, and, lastly, *C. difficile*—which can cause antibiotic-associated diarrhoea, colitis, and pseudomembranous colitis [64]. An external method for *Clostridia* spp. to enter the body is from the consumption of improperly cooked and stored food, in particular, meat and improperly heated canned food [63]. It is possible that *Clostridia* spp. could spread to CBs when encountering soil, contaminated hands, contaminated surfaces, or the mouth; hence, the presence of this species was investigated.

The results presented in Table 4 show that *Salmonella* spp., *E.coli*, Coagulase +ve *Staphylococcus* spp., *Clostridia* spp., and *Legionella* spp. were not found in any of the samples.

The investigation of Coagulase + ve *Staphylococcus* spp. for sample 1 (the freshest CBs of this investigation) was not present in comparison to pilot investigation 1, where it was present in sample 2 (the fresh CBs of that investigation). This could be, because the sample was slightly fresher or that the bacteria were only present within the sample collected. In this investigation, a small count of *Pseudomonas aeruginosa* was found in sample 1. As this species of bacteria is primarily spread by hand, its presence may be due to unclean hands that transfer the bacteria to CBs. A small count of *Enterococcus faecalis* was found in the control sample, which was likely contaminated by the preparation environment, as this bacterium was not present in the control sample of pilot investigation 1.

In sample 2, *Listeria* spp. was detected from the older CBs. *Listeria* spp. is known to survive in waste water and sewerage sludge for a long period of time [65]. This sample may have been contaminated by this species of bacteria, where its characteristics of surviving for a long period of time can be observed.

**Figure 11:**
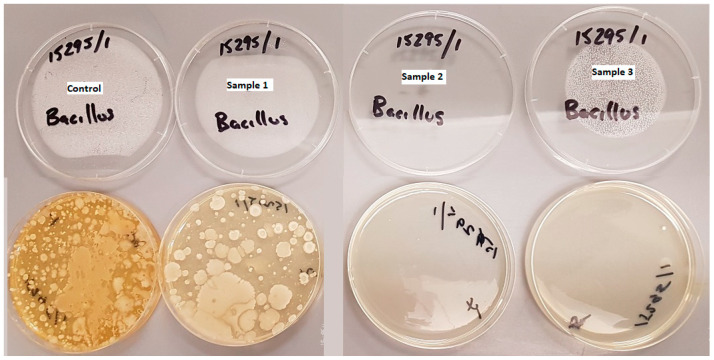
Comparison of *Bacillus* spp. present in samples.

Lastly, for *Bacillus* spp., counts of 70,000 cfu/g were found on control sample 1, 39,000 cfu/g were found on sample 2, and 440 cfu/g were found on sample 3 (Figure 11). These higher quantities are not unusual for soil. As *Bacillus* spp. have been recorded to grow on fresh and cured tobacco leaves, this could be a plausible reason for its presence [66]. When comparing the dried CBs from sample 3 with the other samples, the count of *Bacillus* spp. was significantly lower. This indicates that the majority of *Bacillus* spp. that were present did not survive the drying process. It was noted that sample 4′s preparation method was easier than sample 3′s and it was almost as effective at eliminating the *Bacillus* spp., counts.

The workers dealing with cigarette butts should wear the appropriate PPE and follow OH&S procedures to mitigate the potential risk that the pathogens and chemicals in CBs pose. Some of the PPE recommended include wearing long sleeves and gloves and the use of an appropriate mask.

The results of both pilot investigations 1 and 2 should in no way be interpreted as a comprehensive study. Only six types of bacteria were investigated for pilot investigation 1 and 10 types were investigated for pilot investigation 2. In addition, only a small number of cigarettes were tested. About 75 CBs were collected for each sample for pilot investigation 1, and 150 CBs were collected for each sample for pilot investigation 2. This quantity was then divided to investigate each species of bacteria, thus limiting the scope of the findings.

This paper suggests that further investigation be conducted in this area, particularly given the positive confirmation that *Enterococcus faecalis*, Coagulase + ve *Staphylococcus* spp., *Pseudomonas aeruginosa*, *Listeria* spp., and *Bacillus* spp. were present in such a small population of CBs.

Attempts at determining a way of investigating the presence of viruses failed, as it is impractical to develop a method to utilise for testing and almost impossible to verify whether the produced results would be accurate, or that the virus presented a viable infectious threat.

#### 6.2.3. Presence of Viruses

The possible presence of viruses, such as hepatitis A, B, and C, as well as HIV on CBs, was initially investigated. However, hepatitis A can only be spread through the ingestion of food or water contaminated by the faecal matter of an infected person and is rarely fatal [67]. As a result, it is incredibly unlikely that any CB would be contaminated by the virus. Similarly, hepatitis B has been found in trace amounts in saliva (and, therefore, could contaminate CBs), however this concentration is considered far too insignificant to transmit the virus [68]. Hepatitis C can only be spread through an infected person’s blood directly entering the body of a non-infected person [69]. Hepatitis C can be disregarded as a threat, as CBs will not be ingested or come into contact with any worker’s orifices (as they will be wearing PPE). HIV/AIDS is also a serious virus, which can only be spread through blood-to-blood contact or through contact with an infected person’s various bodily fluids (not including saliva) [70]. As a result, HIV is highly unlikely to be present on CBs, and cannot be spread if handlers are protected with PPE.

### 6.3. Potential Cleaning Methods for CBs

#### 6.3.1. Naphthalene

Naphthalene is a white crystalline powder that produces a well-recognised smell (mothballs). The chemical is isolated from coal tar and has a relatively short half-life in the atmosphere of 3–8 h [71]. Naphthalene is the major active ingredient in ‘toilet cakes’, and it is used as a fumigant [72]. Naphthalene is a suspected carcinogen, and exposure to very high concentrations can lead to damage to blood cells and haemolytic anaemia. However, exposure to low concentrations in the open air should be safe for adults and children older than 3 [73]. Naphthalene and its derivatives have proven antimicrobial capacities [74]. In addition, a common dye, β-naphthol, possesses effective antimicrobial properties [75]. Mkpenie et al. (2008) confirmed this, testing azo-2 naphthol and 2-naphthol on five pathogenic microorganisms: *Escherichia coli*, *Staphylococcus aureus*, *Streptococcus faecalis*, *Pseudomonas aeruginosa*, and *Bacillus subtilis* [76]. The results demonstrated that both chemicals were highly effective at killing all the microorganisms tested. When considering Naphthalene’s short half-life in the atmosphere and antimicrobial abilities, in addition to the ease in which ‘mothballs’ and ‘toilet cakes’ can be located and purchased, these products are potentially a very quick, easy, and cheap method for cleaning CBs, as seen in Section 6.2.2. This paper suggests that toilet cakes or mothballs could be placed into the bags that dry CBs are collected in (i.e., by the collector) and tied. The fumigation effects would then likely kill any possible bacteria. Care should be taken not to inhale the fumes that are released when the bag is opened, as per the reasons stated above. The fumes produced should be minimal if only a few toilet cakes/mothballs are used. This method would be cheap, simple, and effective.

The following methods are more suitable for large scale-cleaning plants.

#### 6.3.2. Ozone

Ozone $\left(O_{3}\right)$ is an unstable gas with powerful oxidising properties. It is predominately produced naturally as a result of ultraviolet rays irradiating oxygen molecules in the stratosphere, where up to 90% of all ozone is located [77]. Research has shown that ozone is an effective antibacterial agent and that it is useful against both gram positive and gram-negative bacteria, as well as spores and vegetative cells [78]. This antimicrobial power is predominately a result of the oxidising potential of ozone, where molecular ozone or its decomposition products, such as radical hydroxyl ions, react with bacteria/virus cells and disrupt normal functions, thereby resulting in inactivation or destruction [79]. Ozone generators are widely available and, therefore, could be considered to be easily accessible in most situations. Ozone has been used to sterilise equipment and containers in the food industry for many years, including in Japan, where the method has been utilised in food manufacturing facilities since 1982 [80]. Using ozone to sanitize surfaces, equipment, and even food is quite safe, as, even though ozone is highly toxic, it quickly disassociates into oxygen [81].

Ozone is effective as an antimicrobial due to its potent oxidisation capabilities [82]. The third oxygen atom in ozone is only loosely bonded and, as a result, is unstable and highly reactive. It will attach itself to organic matter, such as bacteria, and destroy them through the process of oxidisation [83]. Ozone has also been shown to have a capacity to destroy a wide variety of viruses, including hepatitis A and influenza A [84]. Treating fruits and vegetables with ozone has also been shown to increase the shelf life of the products [85,86]. It is essential to remember that ozone is a toxic gas, and it can be lethal in sufficiently high doses [87]. Therefore, it is essential that system designs ensure that humans have no exposure to ozone.

Ozone sterilisation of surfaces can occur in two main forms: gaseous ozone and ozonated water. Objects can be submerged in ozonated water for treatment or can be exposed to continuously generated gas-phase ozone. Rooms can be sealed, whilst gaseous ozone is generated for sterilisation. The ozone will decompose into oxygen atoms at a rate that is influenced by both room temperature and the surface composition of any catalyst [88]. No personnel can be present inside a sealed room during the gaseous ozone sterilisation process for the reasons stated above. A gaseous ozone concentration of 25 parts-per-million (ppm) in air at a Relative Humidity (RH) of 90% was found to be bactericidal after an exposure time of 20 min. [81]. A large volume of research shows that ozone has substantial oxidising, antibacterial, and antiviral capabilities, which suggests that it could be applied for the treatment of any potential bacteria, viruses, and fungal spores on CBs. This paper suggests that gas phase ozone is a more appropriate method when considering the need to control the moisture content of mixes, and the relatively complex process of producing large volumes of ozonated water.

#### 6.3.3. Hydrogen Peroxide

Hydrogen peroxide $\left(H_{2}O_{2}\right)$ is a weak acid with strong oxidising capabilities and, therefore, can be utilised as a powerful bleaching agent [89]. Hydrogen peroxide is commonly utilised as a sterilisation technique in hospital settings, where it is used to disinfect rooms, in addition to re-usable medical and biomedical equipment [90,91]. It is also commonly used in food manufacturing and industrial settings, and, more recently, in water treatment [92]. Hydrogen peroxide quickly decomposes into water and oxygen (its constituent atoms), and is therefore a non-toxic and environmentally friendly replacement for room sterilisation by formaldehyde, which is an expensive and carcinogenic chemical [93]. Hydrogen peroxide can be employed in either a liquid or gaseous form (vaporized hydrogen peroxide). As a liquid, it is commonly used as a surface disinfectant and, in some concentrations, as an antiseptic medicine [92].

Vaporised hydrogen peroxide (VHP) is used for the disinfection of surfaces, re-usable medical equipment, and room fumigation [90,94]. Hydrogen Peroxide, as an oxidising agent, acts by forming HO. Free radicals react with bacteria/virus cells and, thus, prevent the correct functioning of these cells. This results in the inhibition of infection and replication processes [95]. An advantage of vaporised hydrogen peroxide when compared to gaseous ozone is that it has relatively low toxicity to humans and breaks down spontaneously into harmless components [92]. Vaporised hydrogen peroxide generation systems are widely available. Hydrogen peroxide may be an effective treatment method for any viruses, bacteria, or fungi that may be present on CBs as a result of its availability and sterilising properties.

#### 6.3.4. Non-Ionising Ultraviolet Light Radiation

Short wave ultraviolet light (250–260 nm) has been successfully used to inactivate microorganisms in water and food in addition to those that are located on surfaces [96]. Additionally, UVC light has been successfully utilised to disinfect high-touch surfaces in hospital settings [97]. Research also indicates that the effective reduction of bacteria on stainless steel and polypropylene surfaces can be increased by utilising dry heat during the UV sterilisation procedure [98].

UV light comprises wavelengths between 100 nm and 400 nm. These wavelengths are non-ionising. Short wave ultraviolet light (UVC) refers to UV wavelengths of between 200 nm and 280 nm [99]. UVC is commonly termed the ‘germicidal spectrum’, because it is the band width of UV radiation that demonstrates the greatest biocidal effects on bacteria [100]. The lethal effect of UVC light is due to the direct alteration of microbial DNA, resulting from the formation of dimers. Once the DNA is damaged, the cells can no longer reproduce and they are inactivated [101].

Short wave UV lamps are widely available and they are identical to fluorescent lamps; however, they lack a phosphor coating, which allows for the glass to transfer UVC. It is important to note that short wave UV lamps that produce radiation of 260 nm or lower will also produce ozone and, therefore, must be monitored for safety [96]. UV light, predominately UVB, but in some circumstances UVA, can penetrate human skin and cause cyclobutene pyrimidine dimers (mainly thymine dimers), which result in mutations that are found in skin cancers [102]. In contrast, UVC light is absorbed by the outer layer of human skin (epidermis) and it is not known to form CPD (cyclobutane pyrimidine dimers) in human skin (Zeman, ScD, and CHP n.d.). Despite this, exposure of the eye to UVC can cause incredibly painful corneal burns (Zeman, ScD, and CHP n.d.) or result in the development of CPD in the first 10% of the cornea [103]. Therefore, whilst UVC lamps are relatively safe to use, it is essential to ensure that workers are not exposed to UV irradiation systems whilst they are in operation.

#### 6.3.5. Dry and Moist Heat Treatment

Heat treatment for the sterilisation of food in the food industry has been utilised for over 90 years, as well as in healthcare settings [104,105]. There are two heat treatment methods, dry heat sterilisation, and moist heat sterilisation. Dry heat sterilisation involves purely heated air with no or little moisture, which kills cells via oxidisation. Temperatures usually exceed 180 °C and exposure times generally last up to 60 min. [105]. Temperature increases result in a reduced time of exposure required to achieve sterilisation. Alternatively, moist treatment sterilisation involves lower temperatures than dry heat sterilisation and it is applied as steam under pressure, resulting in what is considered as being a generally more efficient sterilisation method, in that highly resistant spores can be destroyed with temperatures of around 121 °C after approximately 30 min. [105]. Dry heat sterilisation requires higher temperatures and longer exposure times because bacteria are far more resistant to dry heat sterilisation than moist heat sterilisation [104]. This is predominately a result of the thermal conductivity of water vs. air, where water allows more heat to be transferred into the bacteria cell.

However, steam will only destroy cells on contact with the surface of an object, whereas radiant heat will still result in cell death [105]. The implications of this are that large volumes of CBs may not be successfully treated with the moist heat sterilisation method, as it is unlikely steam would be able to penetrate/access all surfaces. However, moist heat sterilisation could be utilised if relatively small volumes of CBs are placed in an autoclave at any given time. This would introduce additional handling times (i.e., repetitive loading/unloading of small samples, etc.).

The ignition temperature of paper has been reported to be in the range of 218–246 °C and, therefore, under dry heat temperatures of 180 °C CBs would not be expected to combust [106]. However, great care must be ensured if dry heat sterilisation is utilised for the treatment of CBs, as the presence of various chemicals may alter the ignition temperature.

#### 6.3.6. Notes on Nicotine

Nicotine is the most common chemical found in CBs. It is highly addictive and a known carcinogen. Furthermore, the toxicity of Nicotine does lend it some antimicrobial power, and high concentrations of nicotine, diluted in phosphate-buffered saline, have been found to be effective at killing a number of common bacteria, including *Escherechia coli* [107]. However, the same study found that nicotine had little or no effect on *Staphylococcus aureus*. Similarly, it has been reported that both aqueous and ethanolic extracts of Nicotiana tabacum were effective at inhibiting the growth of M.tubercolosis [108]. Further study found that six extracts of Nicotiana tabacum (Ethanol, Acetone, Butanol, Water, n-Hexane, and Ethyl acetate) were effective in inhibiting the growth of a number of gram positive and gram-negative bacteria, many of which are known pathogens [109].

Historically, tobacco was used to treat several ailments. A review of 128 cases of tobacco treatment, published between 1785 and 1860, identified several allegedly successful applications of the plant to treat a number of ailments. These included the treatment of tetanus, rodent ulcer, general ulcers, and wounds [110]. Additionally, in 1924, a salve composed of burnt tobacco leaves and lanolin was pronounced to have antiseptic effects on superficial ulcers and wounds [111]. Before its introduction to Europe, tobacco was also utilized by the Native American populations. Fernando Ocaranza summarised the medicinal uses of tobacco by Native Americans in Mexico as being for the relief of pain and treatment of wounds and burns [111].

Nicotine is a toxic chemical and lethal to humans. Although the literature generally accepts that a dose of 30–60 mg is lethal, growing evidence supports 0.5 g as being a lethal dose [112].

When considering the findings discussed here, it would be expected that due to the toxicity of nicotine and other chemicals in CBs and CB smoke, that they would present an incredibly hostile environment for bacteria to survive in. However, our initial microbiological pilot study into the presence of bacteria on CBs has shown that bacteria can possibly survive on CBs, albeit for an unknown period. Consequently, further investigations into this area should be undertaken.

### 6.4. Safe Handling of CBs

Prior to discussing the safe handling of CBs, it is known that high levels of dust exposure can lead to respiratory problems, most notably in a brick factory environment where workers are likely to encounter dust particles, which is seen in a large variety of studies conducted across the world. One such study found that exposure to high levels of dust in two brick manufacturing plants in Croatia led to an increased prevalence of chronic respiratory symptoms in exposed workers when compared to the control group [113]. Similar research was conducted in Cape Town, South Africa, where 268 brick workers from five brickworks were studied. It was found that both smoking and exposure to dust had an impact on the prevalence of chronic respiratory disease. However, smoking generally had a weaker effect when compared to dust exposure, and only affected early onset symptoms [114]. Similarly, brick workers in Nepal and Pakistan were found to suffer significant volumes of respiratory problems that were predominately linked to particulate matter exposure [115,116]. Additionally, studies have linked silica exposure in the brick manufacturing industry to silicosis, tuberculosis, and lung cancer. Fishwick et al. (2015) states that current workplace exposure contributes to the risk of developing silicosis [117]. Because it is difficult to control the levels of dust generated within brick manufacturing plants and their environments, it is suggested that employees should always wear protective masks when exposed to dust, particularly where no effort has been made to reduce the volume of dust generated.

It is essential to wear appropriate masks when handling dry CBs. This is to protect against any possible inhalation of VOC or exposure to any pathogens that may be present in the sample, and it is especially important in a brick factory due to the dust exposure alone. Hand protection is required to ensure that pathogens do not enter cuts by chemicals getting absorbed through the skin. In addition to this, it is very important to wear gloves when handling CBs that are moist, as heavy metals, such as lead, cadmium, and arsenic, can leach off the butts.

It is recommended that CBs be sterilised immediately upon or prior to delivery to the manufacturing site. Several appropriate methods have been discussed that have been deemed to be suitable for industrial scale sterilisation of used CBs. For small volumes of CBs, the use of mothballs or toilet cakes that contain Naphthalene can be cost efficient, as, when placed in the bag containing CBs, the Naphthalene will fumigate all or some of the bacteria that may be present (further investigation is recommended in this area). However, this solution is considered to be viable for small volumes and should be used in the interim until a larger scale sterilisation method can be implemented

Previous investigations into the leachates from manufactured CB bricks show that, after firing, only trace amounts of metal leachates were found in bricks with 2.5, 5.0, 7.5, and 10.0% content of CBs [23]. These concentrations were lower than the regulatory limits set by USEPA (1996) and EPAV (2005) [33,34].

Odour from the incorporation of CBs into brick production should also be addressed. The issue stems from VOCs being emitted from CBs and can either be addressed prior to entering the manufacturing site or during manufacturing of CB bricks. It should be noted that odour disappears after the firing of the bricks occurs.

As always, it is essential that local regulations and standards regarding the handling of municipal waste be adhered to.

## 7. Implementation Guide

The following steps may be followed for the implementation of recycling CBs in fired-clay bricks.

- For CB collection, aim to develop a close relationship with CB collection companies to facilitate the delivery of CBs to manufacturing sites. These CBs are normally collected from modern bins or receptacles.
- Once the CBs are collected, a sterilisation method (Section 6) should be used to clean the CBs from bacteria. The odour may be purged from CBs in this stage (Section 5). If mothballs (containing naphthalene) are used, they should be put into the bags containing CBs to inactivate any bacteria that may be present. This can be done by the collector or by workers on-site. Care should be taken to not breathe in fumes when the bags are opened. The CBs will then be stored on-site.
- When ready, CBs can be incorporated into the brick clay mix through a method suggested in Section 5 for incorporation into Automatic Processes and 5.2 for incorporation into Manual Processes. Once the CBs have been incorporated, the remaining steps that are common within the brick manufacturing process can be followed.
- Always ensure that relevant OH&S standards are followed, the correct PPE is worn, and the fumes are not breathed. Refer to Section 5.1.4_5.1.4_Appropriate_PPE, 5.2.3_5.2.3_Appropriate_PPE, and 6.4_6.4._Safe_Handling for more detail on the safe handling of CBs.

## 8. Conclusions

This study has presented and discussed some of the results of an ongoing study on recycling CBs in fired-clay bricks. The results of a CB energy contribution study, bacteriological investigation of CBs, and the properties of bricks incorporating CBs have been discussed. Additionally, the implementation procedure for recycling CBs in bricks and the pre-processing issues of CBs are analysed and discussed. The main findings are summarised and discussed below.

Energy savings: the calorific value of CBs was investigated. It was found that the average gross calorific value of CBs with remnant tobacco was 16.5 MJ/kg. This is a high value when compared to the calorific value of black coal, which is 20 MJ/kg. If 2.5% of all the bricks produced annually around the world include 1% CB content, the energy consumption of the process can be reduced by over 20 billion MJ. This approximately equates to the power used by one million homes every year in the State of Victoria, Australia. Therefore adding 1% CBs (about 20 kg/m^3^) to the brick clay results in significant energy savings, solves the CB pollution problem globally, and tangibly reduces the environmental impact of the brick manufacturing industry, while concurrently providing financial savings to manufacturers through the reduction in energy for firing bricks. Fired-clay bricks are excellent for recycling waste materials. There is a need for a specially dedicated brick factory in every state/country for recycling cigarette butts and other similar waste materials. Incorporation of large amounts of CBs in fired clay bricks is not recommended due to the high calorific value of CBs.

Bacteriological investigations: a serious concern in the recycling of used CBs is that they may be contaminated with bacteria or viruses from contact with people or the environment. As a result, this study undertook two pilot investigations. The presence of ten common bacteria was investigated on unused, fresh, and old CBs. In the first investigation, used CBs that were one week old, and used CBs that were collected on the day of the testing day were compared with an unused control sample. A small colony of Coagulase + ve *Staphylococcus* spp. was detected on the same-day sample. For the second investigation, used CBs that were collected a day before the testing, used old CBs, and used CBs dried at 105 degrees for 6 h were tested and compared with the control sample. The most significant finding from this investigation was that *Listeria* spp. was detected in the used old CB sample and that all used and unused CB samples, except the dried sample, had significant counts of *Bacillus* spp. It was also found that the effect of the contact of naphthalene balls with fresh used CBs on the reduction of *Bacillus* spp., bacteria, was very significant. This study suggests that naphthalene-containing mothballs be used to fumigate bags that were used to collect and store CBs. Further study is recommended in this area. Several suitable sterilisation methods have been reviewed and discussed to address the potential for the presence of pathogens in CBs.

Sterilisation methods: Following the two bacteriological investigations, several industrial sterilisation measures have been studied, including ozone, vaporized hydrogen peroxide, moist heat sterilisation, and dry heat sterilisation, Ultraviolet Light Radiation (UV), and Naphthalene. These methods could be employed to sterilise large volumes of waste materials. Hydrogen peroxide may be an effective and practical sterilising method for any viruses, bacteria, or fungi that may be present on CBs for large scale-cleaning plants.

Properties of bricks incorporating CBs: the compressive strength of 1% CB content bricks with 15.5% and 17.5% moisture content averaged 27.5 MPa and 25.8 MPa, respectively, down from the 43.2 MPa exhibited by the 15% moisture content control bricks. The standard compressive strength for bricks for low-medium rise residential structures in Australia is between 10 and 20 MPa. The decrease in compressive strength as a result of the increase in CB content, establishes a limitation regarding the content of CB that can be incorporated in fired clay bricks. The dry density of the bricks decreased from 2134 kg m^−3^ to 1964 kg m^−3^ (8% reduction). This results in bricks with lower thermal conductivity, which are lighter and therefore reduce the ‘dead-weight’ of potential structures. Lower thermal conductivity can have a significant influence on the energy consumption of a home, as it reduces the need to use air-conditioning systems to adjust the temperature inside the house. The water absorption and initial rate of absorption of the brick increased as expected, due to the reduction in density of the samples. However, the changes were minor and well within acceptable limits. These results confirm the previous results that were published by Mohajerani et al. (2016).

Implementation procedures: three processing methods have been suggested to incorporate the CBs into the automatic brick manufacturing process. Shredding CBs is expected to improve uniformity in the particle size and distribution of CBs within the mix, thereby leading to clay bricks with more uniform properties. However, the shredding of the CBs must be performed in a fully enclosed environment, such as an enclosed industrial shredder. This is necessary, as it keeps the dust and chemicals from spilling out into the environment.

CB collection systems: in Australia, several companies have developed CB disposal systems. These companies provide receptacles for the disposal of CBs. Currently, the collected CBs are placed in landfill or incinerated by the collection company. There are different types of receptacles, including wall-mounted, post-mounted, and free standing.

CB odour: the odour from VOC emissions in CBs can be an issue for recycling CBs in bricks or any other application. This has been discussed and analysed. UV light has the capacity to oxidise and shatter the molecules in VOC elements from CBs to form new molecules that are non-odorous or less odorous. It is possible to deodorize and sterilize CBs at the same time while using a UV light with an appropriate wavelength. This was studied, and UVC or UVV is suggested for this dual treatment. A comprehensive experimental study is recommended in this area.

### Proposal

Approximately six-trillion cigarette butts are littered worldwide every year, resulting in over 1.2 million tonnes of toxic to highly toxic waste contaminating our cities and the environment. Considering the combined risks from hundreds of highly toxic chemicals and possible pathogens in cigarette butts, it is proposed that the littering of this waste anywhere in cities and the environment be strictly prohibited, and that offenders be heavily fined. This strategy should be supported by appropriate education, guidelines, and advertising. Our cities, parks, waterways, beaches, and oceans have been contaminated for many years, with millions of tonnes of unsightly and toxic cigarette butts. Effective and strong laws and guidelines by governments for solving this global pollution problem are overdue and urgently need to be established. This action should be supported by an adequate number of receptacles installed at critical locations in cities and public places by local governments for the collection of CBs for recycling. The sincere, effective, and urgent support and cooperation of smokers, governments, educators, and waste management industries are essential for ending the littering of cigarette butts in the environment.

## Figures and Tables

**Table 1:** Geotechnical Properties of the Brick Soil.

Test/Properties	Standard	Brick Soil
Specific gravity	AS 1289.3.5.1	2.69
Liquid limit (%)	AS 1289.3.1.1	32
Plastic limit (%)	AS 1289.3.2.1	19
Plasticity index (%)	AS 1289.3.1.1	13
Australian soil classification	AS 1726-1993	CL
Optimum moisture content (%)	AS 1289.5.1.1	16
Maximum dry density (Mg/m^3^)	AS 1289.5.1.1	1.78
Organic content (%)	BS 1377-3	1.23

**Table 2:** Properties of manufactured brick samples.

Sample Identification	Moisture Content (%)	Compressive Strength (MPa)	Water Absorption: Cold (%)	Initial Rate of Absorption (kg m^−2^ min^−1^)	Diametric Shrinkage (%)	Height Shrinkage (%)	Average Density (kg m^−3^)	Thermal Conductivity (W m^−1^ K^−1^)
CB (0%) (0 kg m^−3^)	15.0	43.17	8.15	0.31	5.38	6.16	2134.0	1.107
CB (1%) (20 kg m^−3^)	15.5	27.49	10.53	0.47	4.00	4.34	1991.0	0.906
CB (1%) (20 kg m^−3^)	17.5	25.77	11.51	0.39	5.39	5.98	1964.0	0.873

**Table 3:** Preliminary bacteriological test results for used and unused CBs (Pilot investigation 1).

Sample	*Salmonella* spp. /15g	*Escherichia coli* MPN/g	*Pseudomonas aeruginosa* MPN/g	*Enterococcus* spp. MPN/g	Coagulase + ve *Staphylococcus* spp. cfu/g	*Streptococcus* spp. cfu/g
**Control (unused CBs)**	Not Detected	<2	<2	<2	<100	<100
**Sample 1**	Not Detected	<2	<2	2	<100	<100
**Sample 2**	Not Detected	<2	<2	<2	800	<100

Note: the number of CBs in each sample were: control (75), sample 1 (72), sample 2 (71).

**Table 4:** Preliminary bacteriological test results for used, unused, and dried used CBs and CBs with mothballs (Pilot investigation 2).

Sample	*Salmonella* spp. /15g	*Escherichia coli* MPN/g	*Pseudomonas aeruginosa* MPN/g	*Enterococcus* spp. MPN/g	Coagulase + ve *Staphylococcus* spp. cfu/g	*Streptococcus* spp. cfu/g	*Listeria* spp. /25g	*Bacillus* spp. cfu/g	*Clostridia* spp. /g	Total Leginella spp. cfu/g
**Control (unused CBs)**	Not Detected	<2	<2	4	<100	<100	Not Detected	70,000	Not Detected	<1000
**Sample 1**	Not Detected	<2	2	<2	<100	<100	Not Detected	70,000	Not Detected	<1000
**Sample 2** **Stored CBs**	Not Detected	<2	<2	<2	<100	<100	Detected	39,000	Not Detected	<1000
**Sample 3** **Dried** **CBs**	Not Detected	<2	<2	<2	<100	<100	Not Detected	440	Not Detected	<1000
**Sample 4** **with** **Mothballs**	Not Detected	<2	<2	<2	<100	<100	Not Detected	4100	Not Detected	<1000

## Abbreviations

Cigarette butt(s) CB(s); Occupational health and safety OH&S; Personal Protective Equipment PPE; initial rate of water absorption IRA; Acceptable Max Odour Limit AMOL; water content WC, Vaporised hydrogen peroxide VHP; Deoxyribonucleic acid DNA; ultraviolet light UV (UVC short wavelength, UVB medium wave length; UVA long wave length); volatile organic compound(s) VOC(s).

## References

[1] The Tobacco Atlas (2018). ‘Consumption’, American Cancer Society. https://tobaccoatlas.org/topic/consumption/.

[2] Carlozo L.R. (2018). ‘Kicking Butts’, Chicago Tribune. http://www.chicagotribune.com/news/ct-xpm-2008-06-18-0806170174-story.html.

[3] Keep Australia Beautiful (2017). Cigarette Butts. http://www.kabc.wa.gov.au/report-littering/cigarette-butts.

[4] World Health Organization (2017). Tobacco and Its Environmental Impact: An Overview.

[5] Mackay J., Eriksen M., Eriksen M.P. (2002). The Tobacco Atlas.

[6] Srbinoska M., Radojičić V., Đulančić N., Kirkova S. Possibilities for managing the cigarette butts waste. Proceedings of the 26th International Conference Ecological Truth & Environmental Research.

[7] Hoffmann D.H.I. (1997). The Changing Cigarette, 1950–1995. J. Toxicol. Environ. Health.

[8] Australian Government (2013). Cigarettes and Poison. http://www.quitnow.gov.au/internet/quitnow/publishing.nsf/Content/cigarettes-and-poison.

[9] Hecht S.S. (2012). Research Opportunities Related to Establishing Standards for Tobacco Products Under the Family Smoking Prevention and Tobacco Control Act. Nicotine Tob. Res..

[10] Hoffmann D., Hoffmann I., El-Bayoumy K. (2001). The less harmful cigarette: A controversial issue. A tribute to Ernst L. Wynder. Chem. Res. Toxicol..

[11] Hoffmann D., Djordjevic M.V., Brunnemann K.D. (1995). Changes in cigarette design and composition over time and how they influence the yields of smoke constituents. J. Smok. Relat. Disord.

[12] Hon N.-S. (1977). Photodegradation of cellulose acetate fibers. J. Polym. Sci. A Polym. Chem..

[13] Dieng H., Rajasaygar S., Ahmad A.H., Ahmad H., Rawi C.S.M., Zuharah W.F., Satho T., Miake F., Fukumitsu Y., Saad A.R. (2013). Turning cigarette butt waste into an alternative control tool against an insecticide-resistant mosquito vector. Acta Trop..

[14] Marinello S., Lolli F., Gamberini R., Rimini B. (2019). A second life for cigarette butts? A review of recycling solutions. J. Hazard. Mater..

[15] Micevska T., Warne M., Pablo F., Patra R. (2006). ariation in, and causes of, toxicity of cigarette butts to a cladoceran and microtox. Arch. Environ. Contam. Toxicol..

[16] Slaughter E., Gersberg R.M., Watanabe K., Rudolph J., Stransky C., Novotny T.E. (2011). Toxicity of cigarette butts, and their chemical components, to marine and freshwater fish. Tob. Control.

[17] Rebischung F., Chabot L., Biaudet H., Pandard P. (2018). Cigarette butts: A small but hazardous waste, according to European regulation. Waste Manag..

[18] (2017). Clean Up Australia Report. https://www.cleanup.org.au/rubbish-report.

[19] Torkashvand J., Farzadkia M. (2019). A systematic review on cigarette butt management as a hazardous waste and prevalent litter: Control and recycling. Environ. Sci. Pollut. Res..

[20] Kurmus H., Mohajerani A. (2020). The toxicity and valorization options of cigarette butts. Waste Manag..

[21] Kurmus H., Mohajerani A. (2020). Leachate Analysis of Heavy Metals in Cigarette Butts and Bricks Incorporated with Cigarette Butts. Materials.

[22] Mohajerani A., Tanriverdi Y., Nguyen B.T., Wong K.K., Dissanayake H.N., Johnson L., Whitfield D., Thomson G., Alqattan E., Rezaei A. (2017). Physico-mechanical properties of asphalt concrete incorporated with encapsulated cigarette butts. Constr. Build. Mater..

[23] Mohajerani A., Kadir A.A., Larobina L. (2016). A practical proposal for solving the world’s cigarette butt problem: Recycling in fired clay bricks. Waste Manag..

[24] Neil J., Ravinda K.D. (1997). Civil Engineering Materials.

[25] Muñoz Velasco P., Morales Ortíz M.P., Mendívil Giró M.A., Muñoz Velasco L. (2014). Fired clay bricks manufactured by adding wastes as sustainable construction material—A review. Constr. Build. Mater..

[26] Prasertsan S. (1995). Preliminary Study on Brick Making Industry in ASEAN Countries. Final Report (Unpublished).

[27] Australian Standard (2003). AS/NZS 1289.5.1.1. Method 5.1.1.. Methods for Testing Soils for Engineering Purposes—Soil Compaction and Density Tests—Determination of the Dry Density/Moisture Content Relations of a Soil Using Standard Compactive Effort.

[28] Sutcu M., Akkurt S. (2008). AS/NZS 4456.1:2008 (Masonry Units and Segmental Pavers and Flags, 2008).

[29] Eliche-Quesada D., Martínez-Martínez S., Pérez-Villarejo L., Iglesias-Godino F.J., Martínez-García CCorpas-Iglesias F.A. (2012). Valorization of biodiesel production residues in making porous clay brick. Fuel Process. Technol..

[30] Sutcu M., Akkurt S. (2009). The use of recycled paper processing residues in making porous brick with reduced thermal conductivity. Ceram. Int..

[31] Kadir A.A., Mohajerani A. (2008). Possible utilization of cigarette butts in light-weight fired clay bricks. Int. J. Civ. Environ. Eng..

[32] Kadir A.A., Mohajerani A. (2011). Recycling cigarette butts in lightweight fired clay bricks. Constr. Mater..

[33] USEPA (1996). Hazardous Waste Characteristics Scoping Study; Office of Solid Waste.

[34] EPAV (2005). Guidelines for Hazard Classification of Solid Prescribed Industrial Waste.

[35] Midwest Research Institute (MRI) (1997). Brick and Structural Clay Product Manufacturing.

[36] Mortar Industry Association (MIA) (2013). Brick and Block Production.

[37] Ibstock Brick Ltd. (2005). How Clay Bricks Are Made.

[38] Africa Clay Brick Association of South (2015). Clay Brick Technical Guide.

[39] Brick Industry Association (2006). Technical Notes on Brick Construction.

[40] Alternatives Development (2012). Enabling Policies in the Indian Brick Sector-Current Status and Future Trends.

[41] Jrup L. (2003). Hazards of heavy metal contamination. Br. Med. Bull..

[42] Charles S.M., Batterman S.A., Jia C. (2007). Composition and emissions of VOCs in main- and side-stream smoke of research cigarettes. Atmos. Environ..

[43] Martuzevicius D., Prasauskas T., Setyan A., O’connell G., Cahours X., Julien R., Colard S. (2019). Characterization of the Spatial and Temporal Dispersion Differences Between Exhaled E-Cigarette Mist and Cigarette Smoke. Nicotine Tob. Res..

[44] Poppendieck D., Khurshid S., Emmerich S. (2016). Measuring Airborne Emissions from Cigarette Butts: Literature Review and Experimental Plan.

[45] Sherman C., Weaver E. (2016). The Senses: Smell and Taste’, The Dana Alliance for Brain Initiatives. https://www.dana.org/uploadedFiles/Pdfs/Brain-Brief-Senses-Smell-and-Taste-FINAL.pdf.

[46] (2018). Encyclopædia Britannica. https://www.britannica.com/science/methane.

[47] Miljøstyrelsen V.F., Agency D.E.P. (2002). Industrial Odour Control.

[48] Laliberte G. What is the Difference between Odour Concentration and Odour Intensity for Regulators?. http://www.odotech.com/en/odour_concentration_vs_intensity/.

[49] Normand Brais (2017). ‘Garbage Room Odors Remediation’, Sanuvox. https://sanuvox.com/wp-content/uploads/2019/04/OdorControl_EN.pdf.

[50] City of Melbourne (2018). Cigarette Butt Disposal. https://www.melbourne.vic.gov.au/business/waste-recycling/Pages/cigarette-butt-disposal.aspx.

[51] (2018). Centers for Disease Control and Prevention. *E. coli* (*Escherichia coli*). https://www.cdc.gov/ecoli/general/index.html.

[52] SA Health (2017). *Salmonella* Infection—Including Symptoms, Treatment and Prevention. https://www.sahealth.sa.gov.au/wps/wcm/connect/public+content/sa+health+internet/health+topics/health+conditions+prevention+and+treatment/infectious+diseases/salmonella+infection/salmonella+infection+-+including+symptoms+treatment+and+prevention.

[53] Centers for Disease Control and Prevention (2018). *Pseudomonas aeruginosa* in Healthcare Settings. https://www.cdc.gov/hai/organisms/pseudomonas.html.

[54] Fraser S.L. (2018). Enterococcal Infections. https://emedicine.medscape.com/article/216993-overview#a4.

[55] Centers for Disease Control and Prevention (2011). *Staphylococcus aureus* in Healthcare Settings. https://www.cdc.gov/hai/organisms/staph.html.

[56] Healthdirect (2018). Staph Infections. https://www.healthdirect.gov.au/staph-infections.

[57] MedlinePlus (2018). Streptococcal Infections. https://medlineplus.gov/streptococcalinfections.html.

[58] Schmid-Hempel P., Frank S.A. (2007). Pathogenesis, virulence, and infective dose. PLoS Pathog..

[59] Food Standards Australia New Zealand 2013 (2013). Listeria Monocytogenes.

[60] Centers for Disease Control and Prevention (2018). *Legionella* (Legionnaires’ Disease and Pontiac Fever). https://www.cdc.gov/legionella/about/causes-transmission.html.

[61] (2017). *Bacillus* Species. http://www.antimicrobe.org/b82.asp.

[62] Government of UK (2008). Bacillus Species (Food Poisoning). https://www.gov.uk/government/collections/bacillus-species-food-poisoning.

[63] Wells C.L., Wilkins T.D. (1996). *Clostridia*: Sporeforming anaerobic bacilli. Medical Microbiology.

[64] Todar K. Pathogenic *Clostridia*, including Botulism and Tetanus (Page 1). http://textbookofbacteriology.net/clostridia.html.

[65] Garrec N., Picard-Bonnaud F., Pourcher A. (2003). Occurrence of *Listeria* sp. and *L. monocytogenes* in sewage sludge used for land application: Effect of dewatering, liming and storage in tank on survival of *Listeria* species. FEMS Immunol. Med. Microbiol..

[66] Larsson L., Szponar B., Ridha B., Pehrson C., Dutkiewicz J., Krysińska-Traczyk E., Sitkowska J. (2008). Identification of bacterial and fungal components in tobacco and tobacco smoke. Tob. Induc. Dis..

[67] World Health Organisation (2016). Hepatitis A. http://www.who.int/mediacentre/factsheets/fs328/en.

[68] Hepatitis Australia (2015). Transmission of Hepatitis B. http://www.hepatitisaustralia.com/hepatitis-b-facts/transmission.

[69] Centers for Disease Control and Prevention (2016). Hepatitis C Questions and Answers for the Public. https://www.cdc.gov/hepatitis/hcv/cfaq.htm.

[70] Aidsinfo (2016). The Basics of HIV Prevention. https://aidsinfo.nih.gov/education-materials/fact-sheets/20/48/the-basics-of-hiv-prevention.

[71] Buckpitt A., Kephalopoulos S., Koistinen K., Kotzias D., Morawska L., Sagunski H. WHO Guidelines for Indoor Air Quality: Selected Pollutants. https://www.ncbi.nlm.nih.gov/books/NBK138704/.

[72] Bond E. (2007). Manual of Fumigation for Insect Control.

[73] Government of New South Wales Naphthalene in Moth Balls and Toilet Deoderant Cakes. http://www.health.nsw.gov.au/environment/factsheets/Pages/naphthalene.aspx.

[74] Rokade Y., Sayyed R. (2009). Naphthalene derivatives: A new range of antimicrobials with high therapeutic value. Rasayan J. Chem..

[75] Gisvolds W.A. (2004). Textbook of Organic Medicinal and Pharmaceutical Chemistry.

[76] Mkpenie V., Ebong G., Obot I.B., Abasiekong B., Mkpenie V., Ebong G., Obot I., Abasiekong B. (2008). Evaluation of the effect of azo group on the biological activity of 1-(4-methylphenylazo)-2-naphthol. J. Chem..

[77] National Center for Biotechnology Information (2005). ‘Ozone’, PubChem Compound Database. CID=24823. https://pubchem.ncbi.nlm.nih.gov/compound/ozone#section=Top.

[78] Guzel-Seydim Z.B., Greene A.K., Seydim A.C. (2004). Use of ozone in the food industry. LWT Food Sci. Technol..

[79] Khadre M., Yousef A., Kim J.G. (2001). Microbiological aspects of ozone applications in food: A review. J. Food Sci..

[80] Naitou S., Takahara H. (2008). Recent Developments in Food and Agricultural uses of Ozone as an Antimicrobial Agent-Food Packaging Film Sterilizing Machine using Ozone. Ozone Sci. Eng..

[81] Sharma M., Hudson J.B. (2008). Ozone gas is an effective and practical antibacterial agent. Am. J. Infect. Control.

[82] Greene A.K., Few B.K., Serafini J.C. (1993). A Comparison of Ozonation and Chlorination for the Disinfection of Stainless Steel Surfaces. J. Dairy Sci..

[83] (2017). Ozone Oxidation is Nature’s Sanitation Powerhouse. http://www.delozone.com/ozone-technology/about-ozone.php.

[84] Burleson G.R., Murray T., Pollard M. (1975). Inactivation of viruses and bacteria by ozone, with and without sonication. Appl. Microbiol..

[85] Norton J., Charig A., Demoranville I. (1968). Effect of ozone on storage of cranberries. Proceedings of the American Society for Horticultural Science.

[86] Rice R.G., Farquhar J.W., Bollyky L.J. (1982). Review of the applications of ozone for increasing storage times of perishable foods. Ozone Sci. Eng..

[87] Hoof F. (1982). Professional risks associated with ozone. Ozonation Man. Water Waste Water Treat..

[88] Batakliev T., Georgiev V., Anachkov M., Rakovsky S., Zaikov G.E. (2014). Ozone decomposition. Interdiscip. Toxicol..

[89] National Center for Biotechnology Information (2004). ‘Hydrogen Peroxide’, PubChem Compound Database; CID=784. https://pubchem.ncbi.nlm.nih.gov/compound/hydrogen_peroxide.

[90] Chung S., Kern R., Koukol R., Barengoltz J., Cash H. (2008). Vapor hydrogen peroxide as alternative to dry heat microbial reduction. Adv. Space Res..

[91] Fu T.Y., Gent P., Kumar V. (2012). Efficacy, efficiency and safety aspects of hydrogen peroxide vapour and aerosolized hydrogen peroxide room disinfection systems. J. Hosp. Infect..

[92] Linley E., Denyer S.P., McDonnell G., Simons C., Maillard J.Y. (2012). Use of hydrogen peroxide as a biocide: New consideration of its mechanisms of biocidal action. J. Antimicrob. Chemother..

[93] Kahnert A., Seiler P., Stein M., Aze B., McDonnell G., Kaufmann S.H. (2005). Decontamination with vaporized hydrogen peroxide is effective against Mycobacterium tuberculosis. Lett. Appl. Microbiol..

[94] Fichet G., Antloga K., Comoy E., Deslys J., McDonnell G. (2007). Prion inactivation using a new gaseous hydrogen peroxide sterilisation process. J. Hosp. Infect..

[95] Pottage T., Richardson C., Parks S., Walker J.T., Bennett A.M. (2010). Evaluation of hydrogen peroxide gaseous disinfection systems to decontaminate viruses. J. Hosp. Infect..

[96] Bintsis T., Litopoulou-Tzanetaki E., Robinson R.K. (2000). Existing and potential applications of ultraviolet light in the food industry–a critical review. J. Sci. Food Agric..

[97] Rock C., Curless M.S., Nowakowski E., Ross T., Carson K.A., Trexler P., Carroll K., Maragakis L.L. (2016). UV-C Light Disinfection of Carbapenem-Resistant Enterobacteriaceae from High-Touch Surfaces in a Patient Room and Bathroom. Infect. Control Hosp. Epidemiol..

[98] Bae Y.M., Lee S.Y. (2012). Inhibitory Effects of UV Treatment and a Combination of UV and Dry Heat against Pathogens on Stainless Steel and Polypropylene Surfaces. J. Food Sci..

[99] Cutler T.D., Zimmerman J.J. (2011). Ultraviolet irradiation and the mechanisms underlying its inactivation of infectious agents. Anim. Health Res. Rev..

[100] Jagger J. (1967). Introduction to Research in Ultra-Violet Photobiology.

[101] Petersson L.P., Albrecht U.-V., Sedlacek L., Gemein S., Gebel J., Vonberg R.-P. (2014). Portable UV light as an alternative for decontamination. Am. J. Infect. Control.

[102] Angela T., Robert P.S., Antony R.Y. (2011). UVA1 Induces Cyclobutane Pyrimidine Dimers but Not 6-4 Photoproducts in Human Skin In Vivo. J. Investig. Dermatol..

[103] Mallet J.D., Rochette P.J. (2013). Wavelength-dependent ultraviolet induction of cyclobutane pyrimidine dimers in the human cornea. Photochem. Photobiol. Sci..

[104] Smelt J.P., Brul S. (2014). Thermal inactivation of microorganisms. Crit. Rev. Food Sci. Nutr..

[105] Darmady E.M., Hughes K.E., Jones J.D., Prince D., Tuke W. (1961). Sterilization by dry heat. J. Clin. Pathol..

[106] Cafe T. Physical Constants for Investigators. http://www.tcforensic.com.au/docs/article10.html#2.1.

[107] Pavia C.S., Pierre A., Nowakowski J. (2000). Antimicrobial activity of nicotine against a spectrum of bacterial and fungal pathogens. J. Med. Microbiol..

[108] Adeleye I., Onubogu C., Ayolabi C., Isawumi A., Nshiogu M. (2008). Screening of crude extracts of twelve medicinal plants and “wondercure” concoction used in Nigeria unorthodox medicine for activity against mycobacterium tuberculosis isolated from tuberculosis patients sputum. Afr. J. Infect. Dis..

[109] Bakht J., Azra, Shafi M. (2012). Antimicrobial activity of Nicotiana tabacum using different solvents extracts. Pak. J. Bot..

[110] Stewart G.G. (1967). A history of the medicinal use of tobacco 1492–1860. Med. Hist..

[111] Charlton A. (2004). Medicinal uses of tobacco in history. J. R. Soc. Med..

[112] Mayer B. (2014). How much nicotine kills a human? Tracing back the generally accepted lethal dose to dubious self-experiments in the nineteenth century. Arch. Toxicol..

[113] Zuskin E., Mustajbegovic J., Schachter E.N., Kern J., Doko-Jelinic J., Godnic-Cvar J. (1998). Respiratory findings in workers employed in the brick-manufacturing industry. J. Occup. Environ. Med..

[114] Myers J.E., Cornell J.E. (1989). Respiratory health of brickworkers in Cape Town, South Africa: Symptoms, signs and pulmonary function abnormalities. Scand. J. Work Environ. Health.

[115] Raza A., Qamer M., Afsheen S., Adnan M., Naeem S., Atiq M. (2014). Particulate Matter Associated Lung Function Decline in Brick Kiln Workers of Jalalpur Jattan, Pakistan. Pak. J. Zool..

[116] Sanjel S., Khanal S.N., Thygerson S.M., Carter W.S., Johnston J.D., Joshi S.K. (2017). Respiratory symptoms and illnesses related to the concentration of airborne particulate matter among brick kiln workers in Kathmandu valley, Nepal. Ann. Occup. Environ. Med..

[117] Fishwick D., Sumner J., Barber C.M., Robinson E., Codling A., Lewis L., Young C., Warren N. (2015). P61 Respiratory ill health in the silica exposed brick manufacturing sector. Thorax.

